# Cross-national pharmacovigilance of drug-induced intestinal obstruction: disproportionality signals in FAERS and external validation in JADER

**DOI:** 10.3389/fphar.2026.1760208

**Published:** 2026-03-20

**Authors:** Yang Liu, Shengde Wu

**Affiliations:** 1 Department of Urology, Children’s Hospital of Chongqing Medical University, National Clinical Research Center for Children and Adolescents’ Health and Diseases, Ministry of Education Key Laboratory of Child Development and Disorders, Chongqing, China; 2 Chongqing Key Laboratory of Structural Birth Defect and Reconstruction, Chongqing, China

**Keywords:** drug-induced intestinal obstruction, FAERS, JADER, pharmacovigilance, real-world evidence, reporting odds ratio

## Abstract

Using deduplicated FAERS reports from Q1 2004 to Q4 2024 (18,532,100 unique reports), we identified 62,919 cases of drug-induced intestinal obstruction (DIO) and prioritised the 50 suspect drugs with the highest DIO report counts. Disproportionality screening using both the reporting odds ratio (ROR) and Bayesian information component (IC) showed that signal magnitudes varied widely across the Top 50 set (ROR 0.62–5.08; IC −0.69–2.33), with 28/50 drugs meeting prespecified signal criteria by both metrics. Bevacizumab demonstrated the strongest disproportionality (ROR 5.08; IC 2.33), while adalimumab contributed the largest number of DIO reports. Time-to-onset analyses conducted at the report–drug pair level indicated that DIO frequently occurred after weeks to months of exposure (median 100 days; IQR 19–469), with substantial drug-level heterogeneity and a predominance of delayed-onset events (>30 days) for most agents. Cross-database benchmarking in JADER for 20 overlapping drugs showed moderate concordance, supporting partial transportability of leading signals while underscoring setting-specific heterogeneity. Comparison with current product information using a three-level framework (Yes/No/Unclear) suggested incomplete alignment between disproportionality signals and explicit obstruction terminology (12/50 Yes, 28/50 No, 10/50 Unclear). These findings are hypothesis-generating and support sustained clinical vigilance for high-signal therapies, particularly in patients with elevated baseline bowel risk, and confirmation in longitudinal pharmacoepidemiological studies with validated exposure windows and rigorous confounder control.

## 1 Introduction

Intestinal obstruction (IO) is one of the most common and potentially life-threatening surgical emergencies of the gastrointestinal tract, accounting for roughly 15%–20% of hospital admissions for acute abdominal pain and a similar proportion of urgent abdominal operations (Markogiannakis et al., 2007; Catena et al., 2019; Griffiths and Glancy, 2023; Walshaw et al., 2024). Recent analyses of the Global Burden of Disease database indicate that paralytic ileus and IO together cause millions of incident cases and a substantial number of deaths and disability-adjusted life-years worldwide each year, with the greatest absolute burden in older adults and in high-income regions (Proctor et al., 2023; Zhao et al., 2025). In hospital-based series, overall in-hospital mortality for IO typically ranges from about 3% to 10%, but rates increase markedly in complicated cases with strangulation, ischaemia, perforation or sepsis (Markogiannakis et al., 2007; Soressa et al., 2016). The aetiology of IO spans both mechanical and functional forms of obstruction. Mechanical small-bowel obstruction is most often caused by postoperative adhesions, followed by hernias and malignant disease, whereas large-bowel obstruction is usually due to colorectal carcinoma, with volvulus and benign diverticular strictures accounting for most of the remaining cases (Markogiannakis et al., 2007; Catena et al., 2019; Amara et al., 2021; Rajan and Clark, 2022). Functional forms, including adynamic ileus and acute colonic pseudo-obstruction, typically arise after major surgery or in the context of severe systemic illness, electrolyte or metabolic disturbances, or exposure to opioids and other motility-impairing medications, and can be clinically and radiologically indistinguishable from mechanical obstruction (Rami Reddy and Cappell, 2017; Beach and De Jesus, 2023; Griffiths and Glancy, 2023). Although most IO cases are attributable to these structural or postoperative causes, pharmacovigilance studies and clinical reports suggest that a small but clinically important subset is precipitated or aggravated by medications, and such drug-induced forms are likely under-recognised despite being potentially preventable through early identification and timely withdrawal of offending agents (Satake et al., 2021; Handley et al., 2022; Qubad and Bittner, 2023).

Mechanistically, drug-induced intestinal obstruction (DIO) arises when medications precipitate a fixed luminal blockage or induce profound gastrointestinal hypomotility that is clinically indistinguishable from primary mechanical obstruction. On the mechanical side, pharmacovigilance analyses based on the Japanese Adverse Drug Event Report (JADER) database and clinical case series have demonstrated disproportionate reporting and well-documented cases of intestinal obstruction and perforation with agents that promote severe constipation, faecal impaction or the formation of intraluminal concretions, including barium contrast media, ion-exchange resins used to treat hyperkalaemia or hyperphosphataemia, and certain bowel preparations (Tongyoo et al., 2013; Noel et al., 2019; Satake et al., 2021; Wu et al., 2021; Di Rienzo et al., 2024). Chronic exposure to non-steroidal anti-inflammatory drugs can also give rise to “diaphragm disease” of the small bowel and right colon, in which multiple short, fibrotic strictures at sites of drug-induced ulceration result in fixed stenoses, proximal dilatation and, occasionally, caecal perforation (Coolsen et al., 2016; Rahman and Fan, 2017). On the functional side, opioid-induced bowel dysfunction is highly prevalent among long-term opioid users and leads to marked hypomotility, constipation and ileus that may be clinically and radiologically indistinguishable from mechanical obstruction (Camilleri, 2011; Rami Reddy and Cappell, 2017). Strongly anticholinergic psychotropics—particularly clozapine—represent another major cause of gastrointestinal hypomotility, and large pharmacovigilance series have described numerous serious or fatal cases of clozapine-associated constipation, megacolon, ileus and perforation (Every-Palmer and Ellis, 2017; West et al., 2017; Handley et al., 2022). More recently, incretin-based therapies, especially glucagon-like peptide-1 receptor agonists (GLP-1RAs), have generated pharmacovigilance signals for intestinal obstruction or severe ileus, but observational cohorts in patients with type 2 diabetes and inflammatory bowel disease have yielded mixed findings regarding whether these agents truly increase the risk of IO (Sodhi et al., 2023; Nielsen et al., 2025; Gao et al., 2025). At present, however, there is no specific diagnostic category or biomarker for DIO, and attribution to medications relies largely on clinical judgement and the completeness of medication histories. In routine practice, IO cases are often coded simply as “intestinal obstruction”, “ileus” or “pseudo-obstruction” without systematically capturing the temporal relationship to drug exposure, so DIO is likely to be under-recognised and under-reported in clinical series and administrative datasets. Taken together, these diagnostic and coding constraints motivate a system-level pharmacovigilance approach that can screen broadly across therapies, prioritize signals, and triangulate evidence across independent reporting systems and product labels.

The FDA Adverse Event Reporting System (FAERS) is one of the largest spontaneous reporting databases for adverse drug events worldwide and contains extensive post-marketing safety data from 2004 to the present. Maintained by the U.S. Food and Drug Administration (FDA), FAERS collects and analyses reports of adverse drug events (ADEs) and medication errors across a broad range of medicinal products and patient populations, and is widely used for pharmacovigilance signal detection and risk characterisation (Jiang et al., 2014; Satake et al., 2021; Khaleel et al., 2022). Although DIO accounts for only a small proportion of all IO cases, it is associated with substantial morbidity and mortality and, as noted above, is probably under-recognised in routine clinical practice. Previous pharmacovigilance studies of DIO have mainly examined selected drug classes or individual substances within single national or regional datasets and have rarely compared the breadth and magnitude of signals across therapies. To our knowledge, no prior study has combined broad FAERS-based screening with external replication in JADER and a structured label review to contextualize drug-induced intestinal obstruction signals. Such triangulation helps distinguish reproducible signals from those potentially driven by database-specific reporting patterns. Consequently, the overall pattern and relative strength of DIO signals for individual drugs and Anatomical Therapeutic Chemical (ATC) therapeutic classes in a large, global post-marketing database such as FAERS remain poorly defined. Therefore, in this study we used FAERS to systematically identify reports of IO, to quantify and compare disproportionality signals for the most frequently implicated drugs as a hypothesis-generating prioritization and their ATC therapeutic classes using both classical and Bayesian approaches, and to characterise time-to-onset patterns. In addition, we validated key signals in the Japanese Adverse Drug Event Report (JADER) database using a prespecified overlap criterion and conducted a structured label review (Yes/No/Unclear) to provide an integrated pharmacovigilance map to support risk mitigation and earlier recognition of DIO in clinical practice.

## 2 Methods

### 2.1 Data sources and case definition

The data for this study were sourced from two large spontaneous reporting systems: the U.S. Food and Drug Administration (FDA) Adverse Event Reporting System (FAERS) and the Japanese Adverse Drug Event Report (JADER) database. FAERS collects a large volume of information on adverse drug events (ADEs) and medication errors voluntarily submitted by healthcare professionals, manufacturers, and the public (Sakaeda et al., 2013; Iyer et al., 2014; Banda et al., 2016). We downloaded quarterly ASCII data packages from the FAERS website, covering the period from 2004 Q1 to 2024 Q4. These data are organized into seven tables: demographic information (DEMO), adverse event records (REAC), drug usage records (DRUG), event outcomes (OUTC), sources of reports (RPSR), drug therapy duration (THER), and drug indications (INDI) (Iyer et al., 2014; Banda et al., 2016).

Because FAERS is a spontaneous reporting system, there is a risk of duplicate, withdrawn, or superseded reports. To ensure the accuracy of our results, we followed the FDA-recommended deduplication method. We selected the CASEID, FDA_DT, and PRIMARYID fields from the DEMO table and sorted the data by CASEID, FDA_DT, and PRIMARYID in that order. When duplicate CASEIDs were identified, the report with the most recent FDA_DT was retained. If both CASEID and FDA_DT were identical, the report with the largest PRIMARYID was kept (Jiang et al., 2014; Khaleel et al., 2022; Shu et al., 2022). After deduplication, only the most recent version of each case was retained for downstream analyses.

The JADER database is a national spontaneous reporting system maintained by the Pharmaceuticals and Medical Devices Agency (PMDA) in Japan. It contains post-marketing safety reports submitted by healthcare professionals and marketing authorization holders and is publicly available from 2004 onwards (Fujiwara et al., 2016; Tsuchiya et al., 2021). JADER data are provided in four main tables: patient demographics (DEMO), drug information (DRUG), adverse reactions (REAC), and medical history (HIST). We downloaded all available datasets from 2004 Q1 to 2024 Q4 and merged the tables using the case identifiers according to previously published procedures (Barcelos et al., 2019; Tsuchiya et al., 2021; Fusaroli et al., 2024). To improve data quality, we removed records with missing or invalid case identifiers and deleted exact duplicate entries defined as rows with identical case ID, sex, age, reporting year, and reaction preferred terms (PTs) across the merged tables.

All ADEs in both FAERS and JADER are coded using PTs from the Medical Dictionary for Regulatory Activities (MedDRA). In this study, drug-induced intestinal obstruction (DIO) was defined using a pre-specified cluster of MedDRA PTs representing mechanical or functional obstruction of the intestine, including terms such as “intestinal obstruction,” “small intestinal obstruction,” “large intestinal obstruction,” “ileus,” “paralytic ileus,” “gastrointestinal obstruction,” and “intestinal pseudo-obstruction,” consistent with previous pharmacovigilance practice (Bate and Evans, 2009; Cutroneo et al., 2024). The complete PT list used for the DIO definition is provided in Supplementary Table S1. To address potential diagnostic heterogeneity, we additionally pre-specified subtype groupings separating functional ileus/pseudo-obstruction–type PTs from mechanical obstruction PTs for sensitivity analyses (Supplementary Table S2).

Within each database, an individual case safety report (ICSR) was classified as a DIO case if at least one reaction PT matched the DIO cluster and at least one drug was recorded as suspect. In FAERS, we considered drugs coded as primary or secondary suspects in the DRUG table; in JADER, we included drugs coded as “suspected” and excluded those recorded as concomitant or interacting, as recommended in previous JADER-based studies (Fujiwara et al., 2016; Tsuchiya et al., 2021). For all DIO cases, we extracted demographic variables (age, sex), reporting characteristics (reporting country), seriousness outcomes (hospitalization, death, life-threatening, disability, other serious or non-serious), drug indications, and detailed drug information.

To mitigate confounding by indication, particularly for anticancer therapies, we conducted an oncology-indication stratification in FAERS based on the INDI table. Specifically, we mapped each indication MedDRA PT recorded in INDI to its MedDRA System Organ Class (SOC). Reports were classified as Oncology indication if any indication PT mapped to SOC = “Neoplasms benign, malignant and unspecified (incl cysts and polyps)”; otherwise, reports were classified as non-oncology. Reports without usable indication PTs or with unmappable indications were excluded from oncology-stratified disproportionality analyses (Supplementary Table S5).

For the time-to-onset (TTO) analysis in FAERS, because a single report may contain multiple suspect drugs with distinct therapy start dates, we evaluated TTO at the report–drug pair level. We extracted therapy start dates from the THER table and event onset dates from the DEMO table. TTO was defined as the interval in days between the start date of the suspect drug and the onset date of intestinal obstruction. Report–drug pairs with missing start or onset dates, negative intervals (onset earlier than therapy start), or implausibly long intervals (>3,650 days) were excluded from the TTO analysis (Noguchi et al., 2018; Tsuchiya et al., 2021).

**FIGURE 1:**
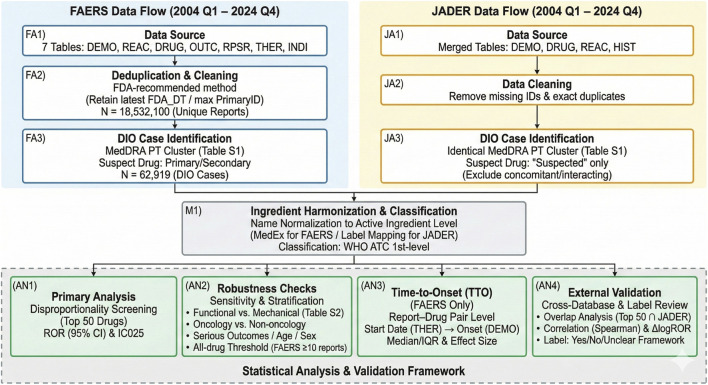
Study design and analytical framework. We integrated FAERS (2004Q1–2024Q4) and JADER (2004Q1–2024Q4) to identify drug-induced intestinal obstruction (DIO) using a prespecified MedDRA PT cluster (Supplementary Table S1), harmonized drug names to the active-ingredient level, and classified drugs by WHO ATC first-level groups. Signals were prioritized among the Top 50 FAERS suspect drugs using disproportionality metrics (ROR and IC025) and evaluated via prespecified sensitivity/stratified analyses (including subtype definitions; Supplementary Table S2), time-to-onset analysis in FAERS at the report–drug pair level, cross-database benchmarking in JADER (Spearman correlation and ΔlogROR), and structured label review (Yes/No/Unclear; accessed 4 February 2026).

The overall process of database cleaning, case selection, and inclusion in the disproportionality and TTO analyses is summarized in a flow diagram (Figure 1).

### 2.2 Drug identification and standardization

Drug names reported in FAERS and JADER lack standardization, as they are submitted by a mixture of healthcare professionals (e.g., physicians and pharmacists) and non-healthcare professionals (e.g., consumers and lawyers). As a result, a wide variety of brand names, generic names, abbreviations, and misspellings may be used. Simply relying on unprocessed product names to identify drug-related adverse events carries a high risk of missing signals.

To minimize this risk in FAERS, we used the Medication Extraction System (MedEx), which has a reported name-normalization accuracy of up to approximately 97% (Xu et al., 2010; Jiang et al., 2014). MedEx (MedEx UIMA 1.3.8, Vanderbilt University, United States) was applied to the DRUG table to standardize reported product names into their corresponding generic ingredient names. Salt forms and common naming variants were harmonized to a single generic ingredient identifier to reduce fragmentation of counts across synonyms. For JADER, Japanese drug names were harmonized to the same set of generic ingredients used for FAERS by cross-referencing the World Health Organization (WHO) Anatomical Therapeutic Chemical (ATC) classification index and information from official product labels (Fujiwara et al., 2016). After normalization, we used the generic name of the active ingredient as the unique identifier for statistical analyses in each database.

In FAERS, we ranked suspect drugs by the number of associated DIO reports and selected the 50 drugs with the highest DIO counts for detailed analysis and presentation. This restriction to the “top 50” was chosen to ensure sufficient cell counts and statistical power for reliable disproportionality and time-to-onset estimates and to facilitate cross-database validation. To mitigate potential selection bias introduced by this presentation-based restriction, we additionally performed a threshold-based sensitivity analysis including all suspect drugs with ≥10 DIO reports, irrespective of ranking (Supplementary Table S7).

**FIGURE 2:**
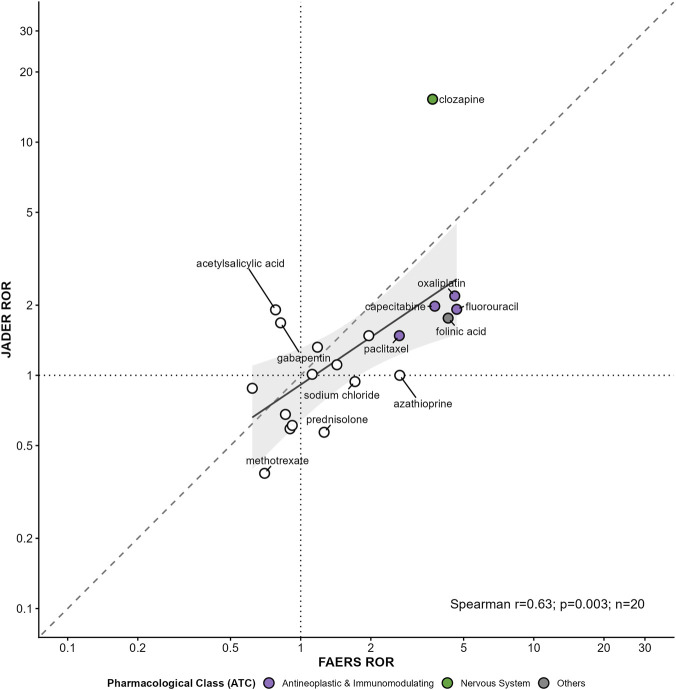
Cross-database validation of disproportionality signals for drug-induced intestinal obstruction (DIO) between FAERS and JADER. Scatter plot of Reporting Odds Ratio (ROR) point estimates for overlapping suspect drugs evaluated in both FAERS and the Japanese JADER database (n = 20). The dashed diagonal line indicates equality (JADER ROR = FAERS ROR), and the dotted reference lines mark the null threshold (ROR = 1.0) in each database. The solid curve shows the LOESS fit with its 95% confidence band (shaded). Points are colored by ATC first-level class. Concordance was assessed using Spearman correlation (r = 0.63, p = 0.003).

**FIGURE 3:**
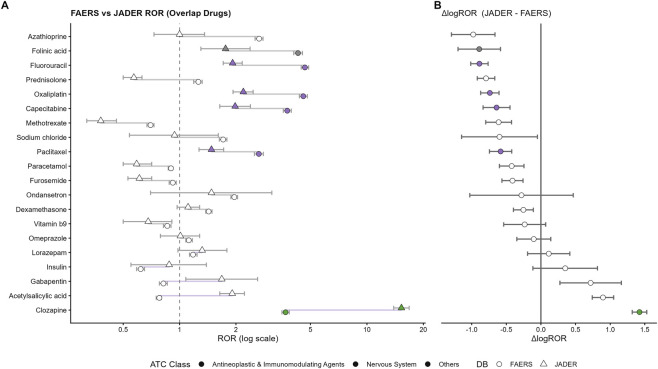
Cross-database comparison of disproportionality signals for drug-induced intestinal obstruction (DIO) between FAERS and JADER. **(A)** Comparative forest plot of Reporting Odds Ratio (ROR) point estimates for the overlapping suspect drugs evaluated in both databases (n = 20), with FAERS shown as circles and JADER as triangles; colors indicate ATC first-level class. **(B)** Drug-level difference in log-transformed ROR between databases (ΔlogROR = logROR_JADER – logROR_FAERS), with error bars indicating the difference for each drug.

In JADER, we independently identified drugs implicated in DIO using the same case definition and performed disproportionality analyses within the database. For cross-database comparison, we prespecified an overlap set defined as drugs that (i) were in the FAERS Top 50 list and (ii) had ≥3 DIO reports in JADER, to avoid unstable estimates from sparse cells. After active-ingredient harmonisation, this overlap set yielded K drugs and was used for cross-database benchmarking. Signal concordance between FAERS and JADER was assessed using Spearman correlation of drug-level logROR point estimates (Caster et al., 2020). To summarise heterogeneity in signal magnitude across databases, we calculated ΔlogROR as logROR(JADER) – logROR(FAERS) (positive values indicating stronger signals in JADER) and visualised these differences using dumbbell plots (Table 5; Figures 2, 3).

To describe pharmacological patterns, we classified the selected drugs using the WHO ATC classification system. Each active substance was assigned to an ATC first-level group (e.g., A: alimentary tract and metabolism; C: cardiovascular system; L: antineoplastic and immunomodulating agents; N: nervous system). For drugs lacking a clear ATC code (such as certain compound preparations or traditional/Kampo medicines), we classified them according to the class or components listed in their official product labels or according to the predominant therapeutic component, following previous approaches (Fujiwara et al., 2016).

To compare pharmacovigilance signals with regulatory information, we systematically reviewed the most recent prescribing information for each of the 50 FAERS study drugs. For products approved in the United States, the latest full prescribing information was retrieved from the Drugs@FDA database; for drugs not marketed in the United States, we consulted European Medicines Agency (EMA) summaries of product characteristics and, where necessary, national regulatory agency websites. We assessed whether intestinal-obstruction–related terms (e.g., “intestinal obstruction,” “bowel obstruction,” “ileus,” “paralytic ileus,” “intestinal pseudo-obstruction”) were explicitly mentioned as adverse reactions, warnings, or precautions. Consistent with previous FAERS-based pharmacovigilance studies that compared detected signals with label information (Hu et al., 2024; Wang et al., 2024; Wen et al., 2024), label evidence was coded using a prespecified three-level scheme (“Yes,” “No,” or “Unclear”) and summarized in Supplementary Table S6. “Unclear” was used when the label contained non-specific gastrointestinal motility wording (e.g., severe constipation or fecal impaction) without explicit obstruction/ileus terminology. All label sources were accessed on 2026-02-04 (Supplementary Table S6).

### 2.3 Statistical analysis

We performed disproportionality analyses to quantify associations between suspect drugs and drug-induced intestinal obstruction (DIO) in FAERS and JADER (Barcelos et al., 2019; Beau-Lejdstrom et al., 2019; Caster et al., 2020; Khouri et al., 2021; Cutroneo et al., 2024). For each drug–event pair, we constructed a 2 × 2 contingency table in the deduplicated reporting universe: (a) DIO reports exposed to the suspect drug, (b) non-DIO reports exposed to the suspect drug, (c) DIO reports unexposed to the suspect drug, and (d) non-DIO reports unexposed to the suspect drug. The reporting odds ratio (ROR) was calculated as (a/c)/(b/d), with 95% confidence intervals (CIs) computed on the log scale using standard errors derived from the four cell counts (Bate et al., 1998; Bate and Evans, 2009). When any cell count was zero, we applied a Haldane–Anscombe continuity correction by adding 0.5 to all four cells before estimating ROR, logROR, and the 95% CI (Bate and Evans, 2009; Sakaeda et al., 2013; Caster et al., 2020; Khouri et al., 2021).

As a complementary Bayesian measure, we estimated the information component (IC) for each drug–event pair and used the lower bound of the 95% credibility interval (IC025) to assess Bayesian signal strength, consistent with established pharmacovigilance practice (Bate et al., 1998; Bate and Evans, 2009). The IC is defined as:

$$IC=\log_{2}\left(\frac{P\left(drug,event\right)}{P\left(drug\right)\times P\left(event\right)}\right)$$

A positive disproportionality signal was defined as drug–event pairs with at least three DIO reports (a ≥ 3) and a lower 95% CI bound of the ROR exceeding 1.0. “Strong signals” were predefined as ROR ≥ 5.0 with at least 10 DIO reports (a ≥ 10). Bayesian signals were considered present when IC025 >0 (Bate and Evans, 2009).

Primary FAERS analyses focused on the 50 suspect drugs with the largest numbers of DIO reports (Top 50 set), to ensure adequate cell counts for stable estimation and for downstream cross-database validation and stratified analyses (Cutroneo et al., 2024). Drug-level results were summarized using ROR/95% CI and IC/credibility intervals and visualized using forest plots and intensity displays. To evaluate temporal stability, we conducted year-stratified analyses and recalculated disproportionality metrics by calendar year from 2004 to 2024 for the Top 50 drugs.

To assess robustness and potential sources of heterogeneity, we conducted a prespecified series of sensitivity, stratified, and subgroup analyses (Supplementary Tables S2–S5, S7, S10; Supplementary Figures S2, S4, S5, S7, S10). First, to address diagnostic heterogeneity in spontaneous coding, we repeated analyses under subtype-specific case definitions by separating functional ileus/pseudo-obstruction–type PTs from mechanical obstruction PTs (Supplementary Table S2). Second, seriousness outcome stratification was performed among reports with available outcome information by recalculating disproportionality metrics within death (yes/no) and hospitalization (yes/no) strata defined using FAERS OUTC coding (Supplementary Table S3). Third, demographic subgroup analyses were conducted by age group and sex using available demographic information, applying the same signal definitions and continuity correction within strata (Supplementary Table S4). Fourth, to mitigate confounding by indication, we conducted oncology versus non-oncology stratified analyses using the INDI-based SOC mapping rule described in Section 2.1; reports without usable or mappable indication PTs were excluded from oncology-stratified analyses (Supplementary Table S5). Fifth, to address potential selection bias introduced by the Top-50 presentation, we conducted a threshold-based sensitivity analysis including all suspect drugs with ≥10 DIO reports irrespective of ranking (Supplementary Table S7).

Using the prespecified overlap set, signal concordance between FAERS and JADER was assessed using Spearman correlation of drug-level logROR point estimates (Caster et al., 2020). To summarise heterogeneity in signal magnitude across databases, we calculated ΔlogROR as logROR(JADER) – logROR(FAERS) and visualised these differences using a dumbbell plot (Table 5; Figure 3).

**FIGURE 4:**
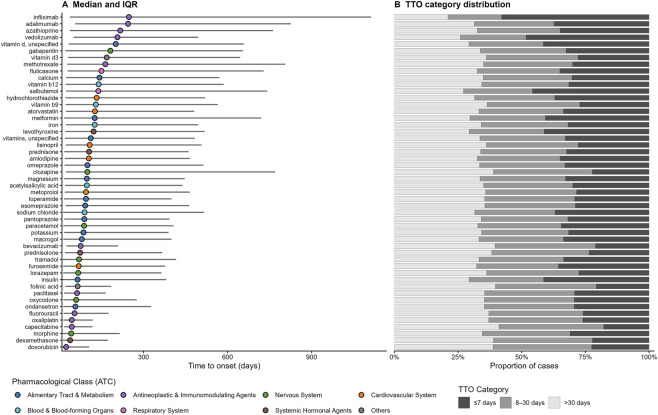
Time-to-onset (TTO) profiles of drug-induced intestinal obstruction (DIO) for the top 50 suspect drugs in FAERS (2004–2024). Analyses were conducted at the report–drug pair level using the evaluable subset with complete and plausible temporal information (n = 46,527 pairs). **(A)** Median TTO (points) and interquartile range (IQR; horizontal bars) for each drug (ordered by median TTO); point colors indicate ATC first-level class. **(B)** Proportional distribution of TTO phases for each drug, categorized as acute (≤7 days), subacute (8–30 days), and delayed (>30 days).

Time-to-onset (TTO) analyses were conducted in FAERS to characterize latency from therapy initiation to event onset. Because a single report may include multiple suspect drugs with distinct therapy start dates, TTO was evaluated at the report–drug pair level. Therapy start dates were extracted from THER and event onset dates from DEMO. TTO was defined as the interval (days) between therapy start and event onset (Noguchi et al., 2018; Tsuchiya et al., 2021). We excluded report–drug pairs with missing therapy start or onset dates, negative intervals (onset preceding therapy start), or implausibly long intervals (>3,650 days). For each Top-50 drug, TTO was summarized using the median and interquartile range (IQR), and phase distributions were categorized as acute (≤7 days), subacute (8–30 days), and delayed (>30 days) (Table 3; Figure 4). To compare latency distributions across ATC first-level classes, we used the Kruskal–Wallis test and reported epsilon-squared (ε^2^) as an effect size (Supplementary Table S8). In addition, we fit quantile (median) regression models to estimate class-specific median ratios relative to the reference category (“Others”), with false discovery rate (FDR) adjustment for multiple comparisons (Supplementary Table S9; Supplementary Figure S8). For class-level modeling, to reduce within-report dependence due to multiple drugs per report, we collapsed TTO data to unique report-by-ATC1 combinations (one representative TTO per report per ATC1, using the earliest available TTO within that class).

Finally, we performed an exploratory drug-combination analysis among commonly co-reported suspect-drug pairs in the Top 50 set. We constructed mutually exclusive exposure groups (A-only, B-only, and A+B) and recalculated disproportionality metrics for co-exposure versus single exposure. A “strict enhancement” pattern was defined when the lower 95% CI bound of the co-exposure ROR exceeded the upper 95% CI bound of the larger single-drug (A-only or B-only) ROR, to conservatively identify combinations with potentially amplified reporting beyond either component alone (Supplementary Table S10; Supplementary Figure S10).

All data processing, statistical analyses, and visualization were performed using R with reproducible scripts, version 4.5.2 (R Foundation for Statistical Computing, Vienna, Austria). A two-sided p-value <0.05 (or false discovery rate-adjusted p < 0.05 where applicable) was considered statistically significant.

## 3 Results

### 3.1 Descriptive analysis in FAERS

From Q1 2004 to Q4 2024, FAERS contained 22,249,476 submitted reports. After FDA-recommended deduplication, the reporting universe comprised 18,532,100 unique reports. Within this deduplicated universe, 62,919 reports met the pre-specified MedDRA PT–based definition of drug-induced intestinal obstruction (DIO) and were included as cases (0.34% of the reporting universe; Supplementary Table S3).

**FIGURE 5:**
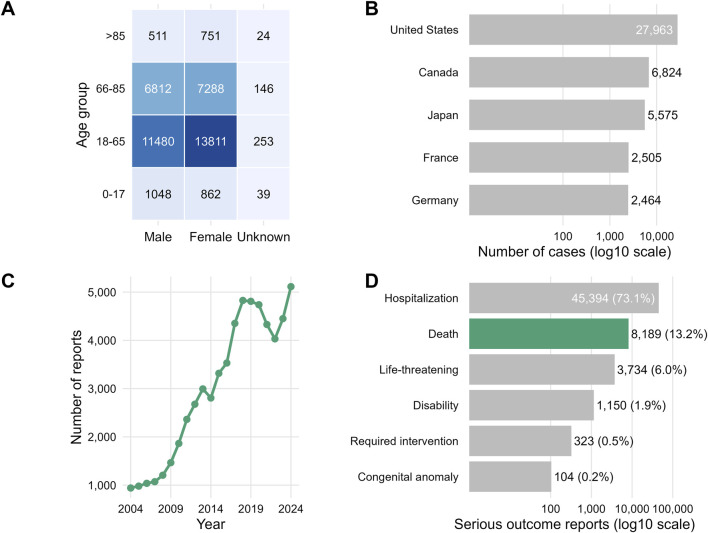
Clinical, demographic, and reporting characteristics of drug-induced intestinal obstruction (DIO) cases in FAERS (2004–2024). **(A)** Heatmap showing the distribution of DIO cases stratified by age group and sex; data are presented for the subset with complete age and sex information (n = 43,025). **(B)** Geographic distribution of reported cases based on the master DIO case set (N = 62,919), highlighting the top five reporting countries and all other countries/regions (including records with missing or unspecified countries, if applicable). **(C)** Yearly trend of DIO case reports submitted to FAERS from 2004 to 2024, increasing from 941 (2004) to 5,113 (2024). **(D)** Distribution of serious outcomes among cases with available outcome information (n = 62,110); outcomes are not mutually exclusive.

Age and sex were jointly available for 43,025 cases (68.4%); therefore, age–sex distributions are reported within this complete-data subset (Figure 5A). Among cases with complete age–sex information, patients aged 18–65 years accounted for 25,544 (59.4%), those aged 66–85 years for 14,246 (33.1%), those aged >85 years for 1,286 (3.0%), and those aged <18 years for 1,949 (4.5%). Within the same subset, females represented 22,712 (52.8%), males 19,851 (46.1%), and sex was coded as unknown in 462 (1.1%) cases (Figure 5A). The remaining 19,894 cases (31.6%) had missing or unspecified age and/or sex.

Geographically, cases were most frequently reported from high-income countries, led by the United States (27,963; 44.5%), Canada (6,824; 10.8%), Japan (5,575; 8.9%), France (2,505; 4.0%), and Germany (2,464; 3.9%); the remaining 17,588 (27.9%) were from other countries or regions (Figure 5B). The annual number of DIO case reports increased steadily from 941 in 2004 to 5,113 in 2024 (Figure 5C).

Seriousness outcomes were available for 62,110 cases (98.7%). Hospitalization was frequently reported (45,410/62,110; 73.1%), and death was also reported in a substantial proportion of cases (8,191/62,110; 13.2%) (Figure 5D; Supplementary Table S3). Overall, DIO case reports in FAERS predominantly involved adult and older patients, were concentrated in high-income reporting regions, and were frequently associated with serious clinical outcomes. Sex differences observed in spontaneous reports should be interpreted cautiously, as they may reflect differential reporting behavior and differences in underlying exposed populations rather than true biological risk differences.

### 3.2 Disproportionality analysis in FAERS

To evaluate drug–DIO associations in FAERS, we performed disproportionality analyses using the reporting odds ratio (ROR) as the primary metric and the Bayesian information component (IC) as a complementary measure. Positive signals were defined as drug–event pairs with at least three DIO reports and a lower 95% confidence bound of the ROR exceeding 1.0. “Strong signals” were predefined as an ROR ≥5.0 with at least 10 DIO reports, and Bayesian signals were considered present when the lower bound of the 95% credibility interval of the IC was >0. To ensure robust cell counts and adequate statistical power for cross-database validation and downstream stratified analyses, our primary analysis focused on the 50 suspect drugs most frequently implicated in DIO.

**FIGURE 6:**
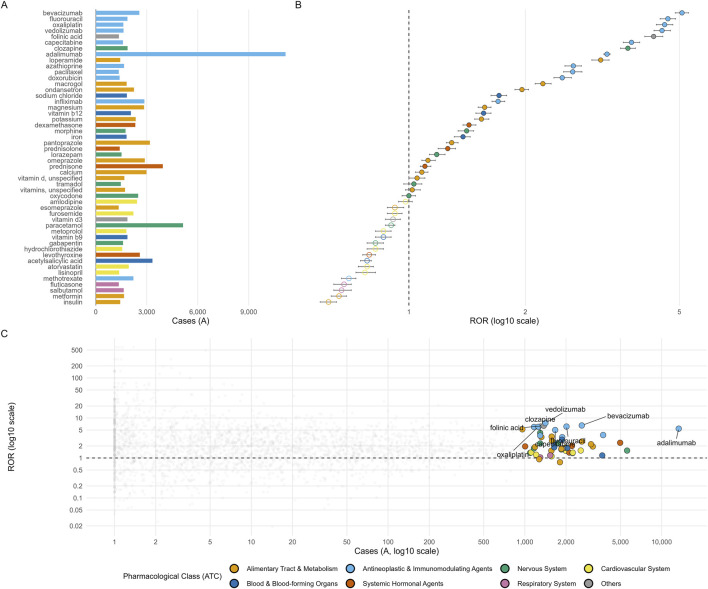
FAERS Top 50 drugs by case count, disproportionality estimates, and selection-bias context for drug-induced intestinal obstruction. **(A)** Number of FAERS intestinal obstruction reports for the Top 50 drugs, colored by pharmacological class (ATC). **(B)** Reporting odds ratios (RORs) with 95% confidence intervals on a log10 scale; the dashed vertical line indicates ROR = 1. Filled symbols denote ROR ≥ 1 and open symbols denote ROR < 1. **(C)** Selection-bias check among all drugs with A ≥ 10: log10(A) versus log10(ROR). Grey points represent all eligible drugs, and colored points highlight the Top 50 drugs using the same ATC classes as in panels A-B. A small jitter was applied on the log10(A) axis to reduce overlap.

Among the top 50 drugs, the number of DIO reports per agent ranged from 1,347 to 11,170. Overall ROR values ranged from 0.62 to 5.08, and IC values ranged from −0.69 to 2.33 (Table 1; Figure 6). Twenty-eight of the 50 drugs met the predefined signal criteria by both ROR and IC (Table 1). Adalimumab contributed the largest absolute number of DIO reports (11,170; ROR 3.25, 95% CI 3.19–3.31), whereas bevacizumab demonstrated the highest disproportionality (ROR 5.08, 95% CI 4.88–5.28; IC 2.33) and was the only drug reaching the strong-signal threshold (Table 1). Other notable positive signals included several antineoplastic, immunomodulating, and gastrointestinal/motility-related agents, such as fluorouracil (ROR 4.67), oxaliplatin (4.58), vedolizumab (4.51), capecitabine (3.76), clozapine (3.68), loperamide (3.13), and macrogol (2.22) (Table 1; Figure 6). In contrast, several widely used medications (including paracetamol, acetylsalicylic acid, metformin, insulin, and fluticasone) yielded RORs below 1.0 with negative or near-zero IC values, indicating no excess reporting of DIO relative to the database background (Table 1).

**TABLE 1:** Disproportionality signals for drug-induced intestinal obstruction (DIO) in FAERS (Q1 2004–Q4 2024): top 50 suspect drugs (ROR, 95% CI, and information component [IC]), with label evidence classification.

Drug	ATC level	ATC category	Number of DIO reports	ROR	95% CI (ROR)	IC	95% CI (IC)	Signal (ROR)	Strong signal (ROR)	Signal (IC)	Package insert suggests risk for intestinal obstruction	Label_support	Label source
Acetylsalicylic acid	B	Blood & blood-forming organs	3336	0.78	0.75–0.80	−0.36	−0.41–-0.31	No	No	No	No	Unclear	Other
Adalimumab	L	Antineoplastic & immunomodulating agents	11170	3.25	3.19–3.31	1.66	1.63–1.69	Yes	No	Yes	No	No	FDA
Amlodipine	C	Cardiovascular system	2425	0.98	0.94–1.02	−0.03	−0.09–0.02	No	No	No	No	No	FDA
Atorvastatin	C	Cardiovascular system	1938	0.78	0.74–0.81	−0.36	−0.42–-0.30	No	No	No	No	No	FDA
Azathioprine	L	Antineoplastic & immunomodulating agents	1658	2.66	2.53–2.79	1.4	1.33–1.47	Yes	No	Yes	No	No	FDA
Bevacizumab	L	Antineoplastic & immunomodulating agents	2564	5.08	4.88–5.28	2.33	2.27–2.38	Yes	Yes	Yes	Yes	Yes	FDA
Calcium	A	Alimentary tract & metabolism	2984	1.08	1.04–1.12	0.11	0.06–0.16	Yes	No	Yes	No	Unclear	Other
Capecitabine	L	Antineoplastic & immunomodulating agents	1594	3.76	3.58–3.95	1.9	1.83–1.97	Yes	No	Yes	No	No	FDA
Clozapine	N	Nervous system	1870	3.68	3.52–3.85	1.87	1.80–1.93	Yes	No	Yes	Yes	Yes	FDA
Dexamethasone	H	Systemic hormonal agents	2326	1.43	1.38–1.49	0.52	0.46–0.58	Yes	No	Yes	No	No	FDA
Doxorubicin	L	Antineoplastic & immunomodulating agents	1400	2.49	2.37–2.63	1.31	1.24–1.39	Yes	No	Yes	No	No	FDA
Esomeprazole	A	Alimentary tract & metabolism	1347	0.92	0.88–0.97	−0.11	−0.19–-0.04	No	No	No	No	Unclear	Other
Fluorouracil	L	Antineoplastic & immunomodulating agents	1865	4.67	4.46–4.89	2.21	2.14–2.28	Yes	No	Yes	No	No	FDA
Fluticasone	R	Respiratory system	1350	0.68	0.64–0.71	−0.56	−0.63–-0.48	No	No	No	No	No	FDA
Folinic acid	Others	Others	1356	4.29	4.07–4.53	2.09	2.01–2.17	Yes	No	Yes	No	No	FDA
Furosemide	C	Cardiovascular system	2210	0.92	0.88–0.96	−0.12	−0.18–-0.06	No	No	No	No	No	FDA
Gabapentin	N	Nervous system	1599	0.82	0.78–0.86	−0.29	−0.36–-0.22	No	No	No	No	No	FDA
Hydrochlorothiazide	C	Cardiovascular system	1555	0.82	0.78–0.86	−0.29	−0.36–-0.22	No	No	No	No	No	FDA
Infliximab	L	Antineoplastic & immunomodulating agents	2857	1.7	1.64–1.77	0.76	0.71–0.81	Yes	No	Yes	Yes	Yes	FDA
Insulin	A	Alimentary tract & metabolism	1433	0.62	0.59–0.65	−0.69	−0.76–-0.61	No	No	No	No	No	FDA
Iron	B	Blood & blood-forming organs	1811	1.38	1.31–1.44	0.46	0.39–0.53	Yes	No	Yes	No	Unclear	Other
Levothyroxine	H	Systemic hormonal agents	2590	0.79	0.76–0.82	−0.33	−0.39–-0.28	No	No	No	No	No	FDA
Lisinopril	C	Cardiovascular system	1378	0.77	0.73–0.82	−0.37	−0.44–-0.29	No	No	No	No	No	FDA
Loperamide	A	Alimentary tract & metabolism	1435	3.13	2.97–3.29	1.64	1.56–1.71	Yes	No	Yes	Yes	Yes	FDA
Lorazepam	N	Nervous system	1511	1.18	1.13–1.24	0.24	0.17–0.32	Yes	No	Yes	No	No	FDA
Macrogol	A	Alimentary tract & metabolism	1813	2.22	2.12–2.33	1.15	1.08–1.21	Yes	No	Yes	Yes	Yes	FDA
Magnesium	A	Alimentary tract & metabolism	2841	1.57	1.51–1.63	0.64	0.59–0.70	Yes	No	Yes	No	Unclear	Other
Metformin	A	Alimentary tract & metabolism	1659	0.66	0.63–0.69	−0.6	−0.67–-0.53	No	No	No	No	No	FDA
Methotrexate	L	Antineoplastic & immunomodulating agents	2214	0.7	0.67–0.73	−0.51	−0.57–-0.45	No	No	No	No	No	FDA
Metoprolol	C	Cardiovascular system	1799	0.86	0.82–0.90	−0.22	−0.29–-0.16	No	No	No	No	No	FDA
Morphine	N	Nervous system	1748	1.41	1.34–1.47	0.49	0.42–0.56	Yes	No	Yes	Yes	Yes	FDA
Omeprazole	A	Alimentary tract & metabolism	2880	1.12	1.08–1.17	0.17	0.11–0.22	Yes	No	Yes	No	Unclear	Other
Ondansetron	A	Alimentary tract & metabolism	2246	1.96	1.88–2.04	0.96	0.90–1.02	Yes	No	Yes	Yes	Yes	FDA
Oxaliplatin	L	Antineoplastic & immunomodulating agents	1619	4.58	4.36–4.81	2.18	2.11–2.26	Yes	No	Yes	Yes	Yes	FDA
Oxycodone	N	Nervous system	2491	1	0.96–1.04	0	−0.06–0.06	No	No	No	Yes	Yes	FDA
Paclitaxel	L	Antineoplastic & immunomodulating agents	1352	2.65	2.51–2.80	1.4	1.32–1.48	Yes	No	Yes	Yes	Yes	FDA
Pantoprazole	A	Alimentary tract & metabolism	3183	1.29	1.25–1.34	0.36	0.31–0.41	Yes	No	Yes	No	Unclear	Other
Paracetamol	N	Nervous system	5134	0.9	0.87–0.92	−0.16	−0.20–-0.12	No	No	No	No	Unclear	Other
Potassium	A	Alimentary tract & metabolism	2349	1.54	1.48–1.61	0.62	0.56–0.68	Yes	No	Yes	Yes	Yes	FDA
Prednisolone	H	Systemic hormonal agents	1405	1.26	1.19–1.32	0.33	0.25–0.40	Yes	No	Yes	No	No	FDA
Prednisone	H	Systemic hormonal agents	3943	1.1	1.07–1.14	0.14	0.09–0.18	Yes	No	Yes	No	No	FDA
Salbutamol	R	Respiratory system	1644	0.67	0.64–0.71	−0.57	−0.64–-0.50	No	No	No	No	No	FDA
Sodium chloride	B	Blood & blood-forming organs	1826	1.71	1.63–1.79	0.77	0.70–0.84	Yes	No	Yes	No	Unclear	Other
Tramadol	N	Nervous system	1470	1.03	0.97–1.08	0.04	−0.04–0.11	No	No	No	Yes	Yes	FDA
Vedolizumab	L	Antineoplastic & immunomodulating agents	1630	4.51	4.30–4.74	2.16	2.09–2.23	Yes	No	Yes	No	No	FDA
Vitamin b12	B	Blood & blood-forming organs	2065	1.56	1.49–1.63	0.64	0.58–0.70	Yes	No	Yes	No	Unclear	Other
Vitamin b9	B	Blood & blood-forming organs	1861	0.86	0.82–0.90	−0.21	−0.28–-0.15	No	No	No	No	No	FDA
Vitamin d, unspecified	A	Alimentary tract & metabolism	1676	1.05	1.00–1.10	0.07	−0.00–0.14	No	No	No	No	No	FDA
Vitamin d3	Others	Others	1864	0.91	0.87–0.95	−0.14	−0.20–-0.07	No	No	No	No	No	FDA
Vitamins, unspecified	A	Alimentary tract & metabolism	1720	1.02	0.98–1.07	0.03	−0.04–0.10	No	No	No	No	No	FDA

**FIGURE 7:**
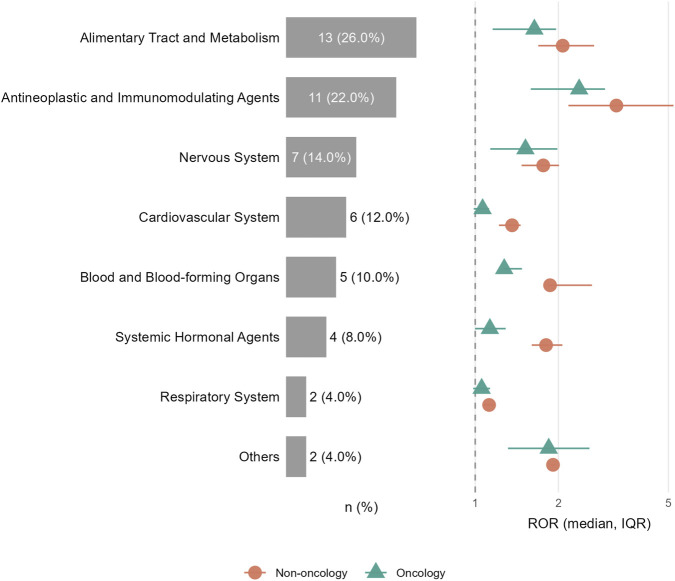
Anatomical Therapeutic Chemical (ATC) classification of the top 50 suspect drugs associated with drug-induced intestinal obstruction (DIO) in FAERS (2004–2024). The pie chart shows the proportional breakdown of ATC first-level categories among the 50 suspect drugs with the highest numbers of DIO case reports in the primary FAERS analysis. ATC A (alimentary tract and metabolism) and ATC L (antineoplastic and immunomodulating agents) were the most represented categories.

When classified by ATC first-level category, the top 50 drugs were predominantly composed of alimentary tract and metabolism agents (ATC A, 26%) and antineoplastic and immunomodulating agents (ATC L, 22%), followed by nervous system drugs (ATC N, 14%), cardiovascular drugs (ATC C, 12%), and blood and blood-forming agents (ATC B, 10%) (Figure 7). Year-stratified analyses further confirmed that elevated RORs for key agents (e.g., bevacizumab, adalimumab, clozapine, vedolizumab, and loperamide) were generally persistent across calendar years from 2004 to 2024, whereas drugs with sub-unity overall RORs remained consistently near-null across the two decades (Table 2).

**TABLE 2:** Year-stratified disproportionality analyses in FAERS for DIO among the top 50 suspect drugs, 2004–2024.

Year	Acetylsalicylic acid	Adalimumab	Amlodipine	Atorvastatin	Azathioprine	Bevacizumab	Calcium	Capecitabine	Clozapine	Dexamethasone	Doxorubicin	Esomeprazole	Fluorouracil	Fluticasone	Folinic acid	Furosemide
2004	0.68 (−0.52)	0.30 (−1.58)	0.99 (0.02)	0.52 (−0.88)	1.33 (0.51)	6.66 (2.76)	1.08 (0.14)	6.85 (2.78)	2.50 (1.36)	1.59 (0.69)	1.89 (0.97)	0.85 (−0.16)	3.39 (1.78)	0.44 (−1.08)	3.80 (1.95)	0.75 (−0.38)
2005	0.67 (−0.55)	0.28 (−1.59)	0.71 (−0.44)	0.72 (−0.45)	0.74 (−0.18)	6.07 (2.61)	1.06 (0.11)	5.64 (2.51)	2.51 (1.37)	1.80 (0.87)	2.84 (1.53)	1.00 (0.05)	3.64 (1.88)	0.61 (−0.65)	3.79 (1.95)	0.90 (−0.13)
2006	0.81 (−0.29)	1.60 (0.79)	1.15 (0.22)	0.67 (−0.54)	1.35 (0.55)	7.35 (2.86)	1.17 (0.25)	6.80 (2.77)	4.18 (2.08)	1.73 (0.81)	1.86 (0.96)	1.00 (0.05)	5.53 (2.47)	0.57 (−0.74)	6.06 (2.61)	0.96 (−0.04)
2007	0.80 (−0.30)	1.49 (0.61)	1.15 (0.23)	0.53 (−0.87)	1.20 (0.40)	6.05 (2.58)	1.26 (0.35)	5.70 (2.51)	3.48 (1.83)	1.67 (0.76)	1.39 (0.54)	0.95 (−0.04)	5.12 (2.36)	0.24 (−1.92)	4.99 (2.34)	0.81 (−0.28)
2008	0.72 (−0.46)	1.14 (0.21)	0.74 (−0.38)	0.66 (−0.56)	1.87 (0.96)	8.87 (3.12)	1.18 (0.25)	6.96 (2.79)	3.16 (1.71)	1.61 (0.71)	2.24 (1.20)	1.36 (0.47)	7.22 (2.84)	0.53 (−0.83)	8.28 (3.05)	0.66 (−0.57)
2009	0.59 (−0.73)	1.21 (0.28)	0.67 (−0.55)	0.87 (−0.18)	2.30 (1.24)	8.43 (3.05)	1.19 (0.26)	7.07 (2.80)	3.00 (1.60)	1.99 (1.00)	2.99 (1.60)	0.84 (−0.21)	6.83 (2.77)	0.46 (−1.07)	6.60 (2.73)	0.88 (−0.16)
2010	0.65 (−0.60)	2.53 (1.34)	0.85 (−0.21)	0.32 (−1.60)	3.21 (1.70)	5.09 (2.34)	1.13 (0.18)	6.29 (2.64)	3.04 (1.62)	2.15 (1.11)	2.35 (1.26)	1.16 (0.22)	4.11 (2.06)	0.55 (−0.83)	4.04 (2.05)	0.94 (−0.08)
2011	0.71 (−0.48)	2.65 (1.39)	0.92 (−0.10)	0.62 (−0.67)	3.35 (1.76)	4.53 (2.18)	1.01 (0.03)	4.74 (2.25)	1.97 (1.00)	1.73 (0.80)	2.71 (1.45)	0.89 (−0.15)	4.10 (2.05)	0.56 (−0.81)	4.14 (2.08)	0.76 (−0.37)
2012	0.87 (−0.18)	2.53 (1.32)	1.23 (0.30)	0.59 (−0.73)	3.52 (1.83)	4.11 (2.04)	0.89 (−0.15)	3.94 (1.98)	3.49 (1.81)	1.82 (0.87)	4.08 (2.03)	0.58 (−0.75)	4.20 (2.08)	0.79 (−0.32)	3.65 (1.89)	0.80 (−0.31)
2013	0.74 (−0.42)	2.47 (1.28)	0.93 (−0.09)	0.66 (−0.58)	4.84 (2.27)	4.92 (2.29)	1.11 (0.15)	3.50 (1.81)	3.46 (1.80)	1.30 (0.39)	1.65 (0.74)	0.66 (−0.57)	4.36 (2.12)	0.96 (−0.04)	5.46 (2.45)	0.84 (−0.23)
2014	0.71 (−0.47)	2.49 (1.28)	0.95 (−0.07)	0.74 (−0.42)	4.28 (2.10)	3.19 (1.68)	0.93 (−0.09)	2.55 (1.37)	3.61 (1.86)	1.40 (0.50)	2.11 (1.09)	0.72 (−0.45)	4.24 (2.08)	0.68 (−0.53)	4.65 (2.23)	0.82 (−0.28)
2015	0.69 (−0.51)	3.52 (1.77)	0.96 (−0.05)	0.71 (−0.47)	4.81 (2.27)	2.84 (1.51)	1.02 (0.03)	2.00 (1.02)	5.17 (2.36)	1.28 (0.36)	2.62 (1.40)	0.87 (−0.17)	4.15 (2.06)	0.57 (−0.78)	4.48 (2.17)	0.75 (−0.40)
2016	0.67 (−0.57)	3.26 (1.64)	0.97 (−0.03)	0.57 (−0.79)	5.07 (2.34)	5.79 (2.52)	0.96 (−0.05)	3.80 (1.93)	3.45 (1.80)	2.02 (1.01)	3.33 (1.74)	1.04 (0.07)	5.05 (2.33)	0.65 (−0.59)	5.13 (2.37)	0.83 (−0.26)
2017	0.66 (−0.59)	7.05 (2.72)	0.84 (−0.25)	0.76 (−0.39)	4.88 (2.28)	4.02 (2.00)	1.02 (0.04)	1.75 (0.83)	2.50 (1.33)	1.33 (0.41)	2.62 (1.40)	1.07 (0.12)	4.13 (2.04)	0.59 (−0.75)	3.94 (1.98)	0.81 (−0.29)
2018	0.65 (−0.61)	5.42 (2.36)	1.01 (0.02)	0.72 (−0.46)	3.90 (1.96)	6.89 (2.76)	1.08 (0.12)	3.62 (1.86)	2.34 (1.24)	1.53 (0.61)	1.75 (0.82)	1.00 (0.02)	4.66 (2.21)	0.59 (−0.74)	4.15 (2.05)	0.74 (−0.42)
2019	0.75 (−0.40)	4.50 (2.11)	0.91 (−0.13)	0.71 (−0.49)	2.90 (1.54)	6.58 (2.70)	1.11 (0.15)	3.94 (1.98)	4.59 (2.19)	1.45 (0.54)	2.28 (1.20)	0.52 (−0.93)	4.26 (2.09)	0.73 (−0.43)	4.25 (2.09)	0.91 (−0.13)
2020	0.71 (−0.48)	3.78 (1.86)	0.89 (−0.15)	0.82 (−0.27)	2.24 (1.17)	4.93 (2.30)	1.22 (0.30)	2.86 (1.52)	3.77 (1.91)	1.04 (0.07)	2.86 (1.52)	0.74 (−0.42)	4.96 (2.31)	0.72 (−0.47)	3.66 (1.88)	0.91 (−0.12)
2021	0.80 (−0.32)	3.92 (1.92)	1.07 (0.11)	1.07 (0.10)	2.51 (1.34)	5.47 (2.45)	1.40 (0.48)	2.63 (1.40)	4.58 (2.19)	1.16 (0.23)	2.50 (1.33)	1.21 (0.29)	4.72 (2.24)	0.82 (−0.27)	3.28 (1.73)	1.01 (0.02)
2022	0.85 (−0.22)	2.15 (1.08)	0.87 (−0.19)	0.83 (−0.26)	1.92 (0.95)	6.02 (2.58)	1.19 (0.26)	3.32 (1.74)	4.02 (2.01)	1.33 (0.42)	3.28 (1.72)	0.82 (−0.26)	4.20 (2.08)	0.73 (−0.44)	3.32 (1.75)	1.02 (0.04)
2023	0.72 (−0.45)	2.91 (1.51)	0.91 (−0.13)	0.88 (−0.17)	2.71 (1.44)	3.48 (1.80)	1.00 (0.00)	2.86 (1.53)	3.67 (1.87)	1.37 (0.46)	2.23 (1.17)	0.74 (−0.41)	4.10 (2.04)	0.70 (−0.50)	3.59 (1.85)	1.10 (0.15)
2024	1.18 (0.24)	2.22 (1.13)	1.34 (0.43)	1.41 (0.49)	1.53 (0.62)	2.55 (1.36)	0.85 (−0.23)	2.72 (1.47)	5.80 (2.53)	1.02 (0.04)	2.18 (1.14)	1.62 (0.71)	4.45 (2.16)	0.89 (−0.16)	3.90 (1.98)	1.23 (0.31)

Gabapentin	Hydrochlorothiazide	Infliximab	Insulin	Iron	Levothyroxine	Lisinopril	Loperamide	Lorazepam	Macrogol	Magnesium	Metformin	Methotrexate	Metoprolol	Morphine	Omeprazole	Ondansetron
0.69 (−0.48)	0.51 (−0.92)	2.06 (1.06)	0.54 (−0.82)	1.60 (0.70)	0.76 (−0.37)	0.31 (−1.57)	5.12 (2.38)	1.45 (0.56)	4.46 (2.20)	2.13 (1.10)	0.42 (−1.15)	0.63 (−0.61)	0.69 (−0.48)	1.88 (0.93)	1.26 (0.36)	2.23 (1.20)
0.74 (−0.39)	0.79 (−0.30)	2.13 (1.12)	0.68 (−0.51)	1.20 (0.31)	0.56 (−0.80)	0.62 (−0.63)	3.20 (1.74)	1.35 (0.46)	1.71 (0.91)	1.60 (0.71)	0.64 (−0.59)	1.15 (0.23)	0.85 (−0.21)	1.77 (0.84)	1.10 (0.16)	3.57 (1.86)
0.74 (−0.39)	0.73 (−0.41)	1.24 (0.38)	0.35 (−1.44)	1.62 (0.72)	0.76 (−0.36)	1.03 (0.07)	6.89 (2.79)	1.48 (0.59)	2.91 (1.60)	1.74 (0.82)	0.25 (−1.92)	0.44 (−1.09)	0.82 (−0.25)	1.79 (0.87)	1.51 (0.61)	3.30 (1.74)
0.83 (−0.22)	0.53 (−0.86)	1.21 (0.33)	0.36 (−1.41)	1.26 (0.38)	0.74 (−0.39)	0.81 (−0.26)	4.38 (2.16)	1.33 (0.44)	4.86 (2.30)	2.28 (1.20)	0.27 (−1.83)	0.56 (−0.77)	1.19 (0.27)	2.39 (1.27)	1.29 (0.38)	1.97 (1.03)
0.78 (−0.29)	0.62 (−0.64)	1.85 (0.92)	0.57 (−0.76)	0.91 (−0.07)	0.80 (−0.30)	0.49 (−0.96)	4.53 (2.21)	1.31 (0.42)	3.01 (1.63)	2.27 (1.19)	0.34 (−1.49)	0.76 (−0.36)	0.98 (0.00)	2.35 (1.25)	1.01 (0.04)	2.55 (1.38)
0.80 (−0.28)	0.59 (−0.73)	1.90 (0.95)	0.57 (−0.77)	1.37 (0.48)	0.71 (−0.47)	0.72 (−0.44)	4.64 (2.23)	1.09 (0.15)	3.06 (1.63)	1.87 (0.91)	0.66 (−0.57)	0.57 (−0.76)	0.83 (−0.24)	2.40 (1.27)	1.30 (0.39)	2.71 (1.45)
0.70 (−0.47)	0.51 (−0.95)	1.77 (0.85)	0.47 (−1.04)	1.48 (0.58)	0.64 (−0.62)	0.73 (−0.42)	5.20 (2.39)	1.12 (0.18)	2.63 (1.41)	1.89 (0.92)	0.51 (−0.93)	0.83 (−0.25)	0.70 (−0.49)	1.52 (0.62)	1.08 (0.12)	1.85 (0.91)
0.78 (−0.34)	0.66 (−0.58)	2.95 (1.56)	0.51 (−0.94)	1.35 (0.45)	0.66 (−0.57)	0.45 (−1.10)	3.23 (1.72)	0.98 (−0.00)	2.35 (1.25)	1.91 (0.93)	0.53 (−0.89)	1.04 (0.07)	0.59 (−0.72)	1.37 (0.47)	1.08 (0.13)	2.28 (1.20)
0.67 (−0.56)	0.60 (−0.70)	2.75 (1.46)	0.72 (−0.45)	1.44 (0.54)	0.75 (−0.39)	0.83 (−0.26)	4.08 (2.04)	1.11 (0.17)	1.54 (0.65)	1.71 (0.77)	0.66 (−0.58)	1.15 (0.22)	0.83 (−0.26)	1.51 (0.61)	0.91 (−0.12)	1.43 (0.54)
0.61 (−0.68)	0.61 (−0.70)	3.24 (1.69)	0.63 (−0.64)	1.26 (0.34)	0.61 (−0.69)	0.62 (−0.67)	2.72 (1.47)	1.16 (0.23)	1.72 (0.80)	1.63 (0.70)	0.52 (−0.91)	0.85 (−0.21)	0.88 (−0.17)	1.34 (0.44)	1.01 (0.02)	1.98 (0.99)
0.69 (−0.51)	0.72 (−0.46)	3.15 (1.66)	0.73 (−0.44)	1.23 (0.31)	0.73 (−0.43)	0.54 (−0.86)	3.01 (1.60)	1.13 (0.19)	1.81 (0.87)	1.47 (0.55)	0.59 (−0.74)	0.87 (−0.19)	0.76 (−0.38)	1.70 (0.78)	1.08 (0.12)	1.76 (0.82)
0.66 (−0.57)	0.56 (−0.81)	2.08 (1.07)	0.35 (−1.49)	1.06 (0.10)	0.77 (−0.36)	0.48 (−1.02)	2.27 (1.20)	1.30 (0.39)	1.91 (0.95)	1.70 (0.76)	0.62 (−0.68)	0.80 (−0.30)	0.70 (−0.49)	1.56 (0.66)	1.14 (0.20)	2.32 (1.21)
0.68 (−0.53)	0.59 (−0.74)	2.62 (1.39)	0.63 (−0.64)	1.29 (0.38)	0.70 (−0.51)	0.71 (−0.48)	2.93 (1.56)	0.97 (−0.02)	1.82 (0.87)	1.73 (0.79)	0.54 (−0.87)	1.04 (0.07)	0.84 (−0.24)	1.27 (0.37)	1.12 (0.17)	2.31 (1.21)
0.76 (−0.39)	0.82 (−0.28)	2.06 (1.05)	0.46 (−1.11)	1.49 (0.59)	0.60 (−0.71)	1.06 (0.10)	2.04 (1.05)	1.07 (0.11)	1.59 (0.67)	1.32 (0.40)	0.58 (−0.77)	0.86 (−0.21)	0.79 (−0.32)	1.94 (0.96)	0.93 (−0.10)	2.00 (1.00)
0.75 (−0.40)	1.29 (0.37)	1.91 (0.94)	0.64 (−0.63)	1.39 (0.48)	0.69 (−0.52)	0.81 (−0.29)	2.81 (1.49)	1.39 (0.49)	1.80 (0.85)	1.56 (0.65)	0.56 (−0.82)	0.79 (−0.32)	0.70 (−0.49)	1.12 (0.18)	0.96 (−0.06)	1.92 (0.94)
0.65 (−0.60)	1.19 (0.25)	1.77 (0.83)	0.54 (−0.87)	1.30 (0.38)	0.84 (−0.24)	0.75 (−0.40)	2.19 (1.15)	1.11 (0.16)	2.55 (1.35)	1.26 (0.34)	0.55 (−0.85)	0.62 (−0.67)	0.87 (−0.18)	2.03 (1.02)	0.86 (−0.20)	1.73 (0.79)
0.78 (−0.34)	0.66 (−0.57)	1.47 (0.56)	0.62 (−0.67)	1.18 (0.25)	0.83 (−0.26)	1.06 (0.10)	3.52 (1.82)	1.65 (0.73)	2.56 (1.36)	1.35 (0.45)	0.67 (−0.56)	0.50 (−0.97)	0.87 (−0.19)	1.34 (0.43)	1.20 (0.26)	2.18 (1.12)
0.98 (−0.02)	1.10 (0.15)	1.41 (0.50)	0.63 (−0.65)	1.42 (0.52)	1.00 (0.01)	0.90 (−0.14)	3.62 (1.86)	1.13 (0.20)	2.55 (1.35)	1.76 (0.82)	0.92 (−0.11)	0.58 (−0.76)	0.89 (−0.16)	0.80 (−0.31)	1.25 (0.33)	1.58 (0.66)
1.25 (0.33)	0.76 (−0.38)	1.42 (0.50)	0.56 (−0.82)	1.49 (0.58)	0.87 (−0.19)	0.61 (−0.68)	3.15 (1.66)	1.00 (0.03)	3.32 (1.73)	1.48 (0.58)	0.67 (−0.56)	0.63 (−0.65)	0.86 (−0.19)	0.86 (−0.20)	1.32 (0.41)	1.64 (0.72)
1.03 (0.06)	0.58 (−0.75)	2.25 (1.15)	0.74 (−0.41)	1.36 (0.46)	0.86 (−0.20)	0.70 (−0.48)	3.30 (1.72)	1.02 (0.05)	1.70 (0.78)	1.19 (0.26)	0.78 (−0.35)	0.85 (−0.22)	0.89 (−0.15)	1.40 (0.50)	0.96 (−0.05)	1.80 (0.85)
1.17 (0.23)	1.17 (0.24)	1.59 (0.66)	1.25 (0.33)	1.68 (0.75)	1.12 (0.17)	0.86 (−0.19)	3.36 (1.75)	0.76 (−0.39)	2.92 (1.54)	1.11 (0.16)	1.54 (0.63)	0.67 (−0.56)	0.95 (−0.05)	1.29 (0.38)	1.35 (0.44)	2.02 (1.02)

Oxaliplatin	Oxycodone	Paclitaxel	Pantoprazole	Paracetamol	Potassium	Prednisolone	Prednisone	Salbutamol	Sodium chloride	Tramadol	Vitamin b12	Vitamin b9	Vitamin d, unspecified	Vitamin d3	Vitamins, unspecified	Vedolizumab
5.51 (2.46)	1.15 (0.22)	3.76 (1.93)	2.10 (1.09)	1.01 (0.03)	1.16 (0.24)	0.93 (−0.04)	1.06 (0.10)	0.73 (−0.41)	1.52 (0.73)	1.00 (0.06)	1.56 (0.69)	0.93 (−0.07)	1.36 (0.53)	1.16 (0.35)	1.04 (0.08)	—
4.29 (2.12)	0.94 (−0.06)	3.08 (1.65)	1.53 (0.64)	1.02 (0.04)	1.29 (0.39)	1.18 (0.30)	1.10 (0.16)	0.71 (−0.44)	1.55 (0.76)	1.31 (0.43)	1.10 (0.20)	1.05 (0.11)	0.63 (−0.50)	0.93 (0.06)	1.08 (0.14)	—
7.59 (2.91)	1.27 (0.36)	2.39 (1.31)	1.38 (0.49)	1.01 (0.02)	1.07 (0.14)	1.09 (0.18)	0.86 (−0.18)	1.02 (0.06)	1.49 (0.68)	1.14 (0.24)	1.04 (0.13)	1.00 (0.04)	1.22 (0.38)	1.01 (0.17)	0.96 (−0.02)	—
5.34 (2.43)	0.89 (−0.13)	3.03 (1.63)	2.20 (1.15)	1.08 (0.12)	1.17 (0.25)	1.00 (0.05)	1.00 (0.02)	0.92 (−0.07)	3.29 (1.75)	1.03 (0.11)	1.35 (0.49)	1.26 (0.36)	0.77 (−0.25)	1.14 (0.31)	0.77 (−0.34)	—
6.97 (2.80)	1.80 (0.86)	4.75 (2.25)	1.37 (0.48)	1.02 (0.05)	1.08 (0.15)	1.22 (0.32)	1.11 (0.17)	0.66 (−0.54)	2.40 (1.30)	0.89 (−0.10)	1.55 (0.67)	0.93 (−0.06)	0.78 (−0.26)	0.78 (−0.22)	1.08 (0.13)	—
8.20 (3.03)	1.39 (0.49)	3.16 (1.68)	0.99 (0.01)	0.93 (−0.10)	1.45 (0.55)	1.33 (0.44)	1.07 (0.11)	0.77 (−0.35)	1.76 (0.85)	0.86 (−0.17)	1.26 (0.37)	0.74 (−0.40)	0.78 (−0.31)	0.73 (−0.36)	1.01 (0.03)	—
7.70 (2.94)	1.46 (0.56)	2.73 (1.47)	1.33 (0.42)	0.91 (−0.12)	1.53 (0.63)	1.19 (0.28)	1.12 (0.17)	0.85 (−0.21)	2.33 (1.23)	0.91 (−0.09)	1.43 (0.53)	0.98 (−0.00)	0.83 (−0.23)	1.28 (0.40)	0.93 (−0.08)	—
6.34 (2.67)	1.67 (0.74)	3.00 (1.60)	1.23 (0.31)	0.90 (−0.14)	1.31 (0.39)	1.70 (0.78)	1.30 (0.38)	0.79 (−0.31)	2.01 (1.01)	1.01 (0.04)	1.70 (0.77)	1.00 (0.02)	0.95 (−0.05)	0.66 (−0.53)	1.03 (0.06)	—
5.90 (2.56)	1.36 (0.45)	2.23 (1.19)	1.05 (0.09)	0.80 (−0.30)	1.00 (0.01)	1.98 (0.99)	1.55 (0.63)	0.62 (−0.66)	1.58 (0.68)	0.93 (−0.08)	1.54 (0.64)	0.90 (−0.14)	0.72 (−0.45)	0.85 (−0.20)	1.13 (0.19)	22.63 (5.85)
4.20 (2.08)	1.20 (0.28)	2.86 (1.52)	1.58 (0.67)	0.89 (−0.16)	1.25 (0.34)	1.05 (0.10)	1.44 (0.52)	0.51 (−0.93)	1.51 (0.60)	1.01 (0.04)	1.36 (0.45)	0.99 (0.00)	0.94 (−0.08)	1.03 (0.06)	0.90 (−0.13)	—
5.53 (2.47)	1.14 (0.19)	1.80 (0.87)	1.79 (0.84)	0.91 (−0.13)	1.53 (0.62)	1.76 (0.83)	1.43 (0.51)	0.73 (−0.43)	1.40 (0.49)	1.18 (0.26)	1.58 (0.67)	1.02 (0.04)	0.78 (−0.33)	0.94 (−0.07)	0.88 (−0.17)	13.38 (3.93)
3.68 (1.89)	1.51 (0.60)	3.12 (1.65)	1.90 (0.93)	0.93 (−0.10)	1.37 (0.46)	1.20 (0.29)	1.46 (0.55)	0.87 (−0.18)	1.86 (0.90)	1.14 (0.20)	1.28 (0.37)	0.93 (−0.08)	0.84 (−0.24)	1.27 (0.36)	0.81 (−0.29)	4.16 (2.13)
4.55 (2.19)	1.47 (0.56)	3.22 (1.70)	1.92 (0.94)	0.96 (−0.05)	1.22 (0.29)	1.22 (0.31)	1.31 (0.39)	0.59 (−0.73)	2.04 (1.03)	1.00 (0.01)	1.24 (0.32)	1.00 (0.02)	0.84 (−0.24)	0.79 (−0.32)	0.66 (−0.58)	5.37 (2.44)
3.58 (1.85)	1.34 (0.42)	2.31 (1.22)	1.45 (0.54)	1.04 (0.06)	1.44 (0.53)	1.11 (0.17)	1.36 (0.45)	0.63 (−0.64)	1.31 (0.40)	1.17 (0.24)	1.43 (0.52)	1.03 (0.05)	1.01 (0.02)	0.87 (−0.19)	0.94 (−0.08)	7.47 (2.89)
4.85 (2.27)	0.91 (−0.13)	2.93 (1.55)	1.13 (0.18)	0.84 (−0.25)	1.61 (0.69)	1.47 (0.56)	1.16 (0.22)	0.58 (−0.78)	1.64 (0.73)	1.14 (0.20)	1.64 (0.72)	1.00 (0.01)	1.08 (0.12)	1.07 (0.11)	0.88 (−0.18)	6.58 (2.71)
3.54 (1.83)	1.68 (0.75)	2.58 (1.37)	0.99 (−0.01)	0.94 (−0.09)	2.07 (1.05)	0.88 (−0.16)	1.10 (0.14)	0.54 (−0.88)	1.82 (0.87)	1.22 (0.30)	1.51 (0.60)	1.05 (0.09)	1.03 (0.06)	1.08 (0.12)	0.85 (−0.22)	5.19 (2.38)
3.60 (1.86)	1.15 (0.20)	2.41 (1.28)	1.28 (0.35)	0.98 (−0.02)	1.77 (0.83)	0.99 (0.01)	1.04 (0.06)	0.79 (−0.32)	2.03 (1.03)	1.07 (0.11)	1.50 (0.59)	0.76 (−0.38)	1.24 (0.31)	1.10 (0.15)	1.05 (0.08)	6.70 (2.74)
4.32 (2.12)	0.61 (−0.70)	2.57 (1.37)	1.46 (0.55)	0.77 (−0.37)	2.03 (1.02)	1.10 (0.16)	0.87 (−0.19)	0.64 (−0.63)	1.74 (0.81)	0.85 (−0.23)	2.08 (1.06)	0.88 (−0.18)	1.24 (0.32)	1.03 (0.05)	1.11 (0.17)	5.74 (2.51)
3.90 (1.97)	0.50 (−0.99)	3.28 (1.72)	0.98 (−0.03)	0.91 (−0.12)	1.64 (0.72)	1.00 (0.01)	0.97 (−0.03)	0.63 (−0.65)	1.73 (0.80)	0.95 (−0.06)	1.59 (0.68)	0.57 (−0.78)	1.09 (0.14)	0.92 (−0.11)	1.22 (0.30)	5.27 (2.39)
3.51 (1.82)	0.71 (−0.48)	1.27 (0.38)	1.53 (0.61)	0.80 (−0.31)	1.43 (0.53)	1.83 (0.88)	1.22 (0.28)	0.67 (−0.56)	1.29 (0.38)	0.95 (−0.05)	2.13 (1.09)	0.69 (−0.52)	1.18 (0.26)	0.94 (−0.09)	1.00 (0.02)	5.24 (2.37)
3.22 (1.70)	0.58 (−0.76)	1.89 (0.94)	1.22 (0.29)	0.83 (−0.26)	1.67 (0.74)	1.18 (0.25)	1.11 (0.15)	0.68 (−0.53)	1.59 (0.67)	0.97 (−0.03)	1.67 (0.75)	0.65 (−0.61)	1.99 (0.99)	0.86 (−0.21)	1.20 (0.28)	5.00 (2.30)

To rigorously address potential selection bias, diagnostic heterogeneity, and confounding by indication, we conducted a comprehensive series of sensitivity, stratification, and subgroup analyses, which are reported in Section 3.3 (Supplementary Tables S2–S5, S7, S10).

### 3.3 Sensitivity, stratification, and subgroup analyses

To evaluate robustness and potential sources of heterogeneity, we conducted a series of prespecified sensitivity, stratified, and subgroup analyses (Supplementary Tables S2–S5, S7, S10; Supplementary Figures S2, S4, S5, S7, S10).

First, case-definition sensitivity analyses separating functional ileus/pseudo-obstruction from mechanical obstruction showed that the overall signal landscape was broadly consistent, while a subset of drugs exhibited preferential signals in one subtype versus the other. In our data, clozapine and morphine showed markedly stronger signals for functional ileus, whereas antineoplastic agents such as fluorouracil more prominently contributed to mechanical obstruction signals (Supplementary Table S2; Supplementary Figure S2).

Second, serious-outcome–stratified disproportionality analyses (death and hospitalization) yielded generally concordant signal patterns with the primary analysis, supporting that findings were not restricted to non-serious reports (Supplementary Table S3).

Third, age- and sex-stratified analyses indicated that most leading signals were robust across strata, with only limited drug-level variations observed across demographic subgroups (Supplementary Table S4; Supplementary Figure S4).

Fourth, to address confounding by indication, oncology vs. non-oncology stratification showed that key signals among antineoplastic and immunomodulating agents remained detectable across strata, with effect sizes generally stronger within oncology-associated reports (Supplementary Table S5; Supplementary Figure S5).

Fifth, to mitigate potential selection bias from the Top-50 presentation, an all-drug threshold analysis including all suspect drugs with ≥10 DIO reports (1,382 eligible drugs; 62,779 cases) showed a consistent pattern of prioritized therapeutic classes and leading agents (Supplementary Table S7; Supplementary Figure S7).

Finally, exploratory drug-combination analyses suggested potentially enhanced reporting for selected co-administered regimens (e.g., fluorouracil plus bevacizumab or capecitabine plus folinic acid) compared with each component alone, warranting further investigation (Supplementary Table S10; Supplementary Figure S10).

### 3.4 Time-to-onset analysis in FAERS

To characterize latency patterns of DIO after drug initiation, we conducted a time-to-onset (TTO) analysis for the same 50 study drugs in FAERS. This analysis was based on the master DIO case set (N = 62,919 unique reports; Section 3.1). Because a single case report may involve multiple co-prescribed suspect drugs with distinct initiation dates, TTO was evaluated at the report–drug pair level.

Among 106,660 DIO report–drug pairs for the Top 50 set, 54,371 pairs lacked usable therapy start or event onset dates, and 5,762 pairs had implausible intervals (onset preceding therapy start or >3,650 days); thus, 46,527 pairs (43.6%) were retained for subsequent TTO analyses (Supplementary Figure S9; Supplementary Table S9). Across the retained pairs, DIO showed a predominantly delayed-onset profile, with a median TTO of 100 days (IQR 19–469) and a P10–P90 range of 3–1,459 days (Supplementary Table S8).

At the individual-drug level, median TTOs spanned an order-of-magnitude range, from 24 days for doxorubicin (IQR 10–106) to 248 days for infliximab (IQR 37–1,112), with wide interquartile ranges indicating substantial variability (Table 3; Figure 4A). Late-onset patterns were most evident for biologics and immunomodulators, including infliximab (median 248 days; 76.1% with TTO >30 days), adalimumab (245 days; 82.7%), vedolizumab (207 days; 82.8%), and azathioprine (217 days; 77.5%) (Table 3; Figure 4B). In contrast, relatively earlier onset was observed for cytotoxic agents, opioids, and corticosteroids, such as doxorubicin (median 24 days; 55.7% within 30 days), dexamethasone (38 days; 45.3% within 30 days), morphine (42 days; 43.3% within 30 days), and fluorouracil (54 days; 35.7% within 30 days), although events occurring beyond 30 days still predominated for most drugs (Table 3; Figure 4).

**TABLE 3:** Time-to-onset (TTO) of DIO in FAERS among the top 50 suspect drugs: report–drug pair–level analysis (median, IQR, and onset-phase distribution).

Drug	Number of DIO reports (TTO-eligible)	Number of DIO reports with usable TTO	TTO coverage (%)	Median TTO (days)	Q1 (days)	Q3 (days)	TTO IQR (days)	TTO <= 7 days (%)	TTO 8-30 days (%)	TTO >30 days (%)
Adalimumab	10866	2310	21.3	245	56	826	770	9.4	7.9	82.7
Paracetamol	4872	2299	47.2	88	14	407	393	17.3	16.4	66.2
Pantoprazole	2995	1497	50.0	89	17	393	376	16.2	17.3	66.5
Acetylsalicylic acid	3194	1467	45.9	98	20	439	419	14.4	16.8	68.8
Prednisone	3780	1362	36.0	106	17	461	444	15.9	16.6	67.5
Bevacizumab	2428	1306	53.8	76	25	210	185	9.7	18.2	72.1
Magnesium	2729	1284	47.1	98	17	447	430	16.0	16.4	67.6
Calcium	2851	1279	44.9	143	24	571	547	13.3	15.3	71.4
Dexamethasone	2244	1239	55.2	38	13	173	160	16.5	28.8	54.7
Omeprazole	2720	1220	44.9	100	18	514	496	15.7	15.9	68.4
Ondansetron	2114	1185	56.1	57	14	327	313	17.0	20.4	62.5
Amlodipine	2297	1128	49.1	105	19	466	447	15.8	14.7	69.5
Potassium	2203	1119	50.8	86	17	389	372	16.0	17.3	66.7
Oxycodone	2361	1078	45.7	60	14	276	262	17.5	21.0	61.5
Levothyroxine	2477	1035	41.8	122	21	518	497	17.1	12.2	70.7
Furosemide	2082	951	45.7	69	14	377	363	18.9	17.1	63.9
Infliximab	2756	940	34.1	248	37	1112	1075	17.6	6.4	76.1
Fluorouracil	1767	886	50.1	54	15	175	160	14.7	21.0	64.3
Vitamin b12	1966	848	43.1	140	22	586	564	13.9	15.1	71.0
Vitamin d3	1783	837	46.9	169	31	645	614	10.9	14.1	75.0
Vedolizumab	1582	833	52.7	207	50	495	445	11.2	6.0	82.8
Iron	1730	807	46.6	126	24	495	471	13.9	14.9	71.3
Atorvastatin	1814	799	44.0	127	23	481	458	14.8	14.6	70.6
Oxaliplatin	1524	791	51.9	44	14	119	105	16.4	23.3	60.3
Vitamin b9	1777	771	43.4	130	22	565	543	12.5	16.7	70.8
Macrogol	1682	766	45.5	80	14	400	386	17.8	17.6	64.6
Vitamins, unspecified	1631	761	46.7	112	19	483	464	15.2	15.5	69.3
Capecitabine	1510	740	49.0	44	17	118	101	12.4	28.6	58.9
Sodium chloride	1732	738	42.6	90	13	516	503	19.2	16.4	64.4
Morphine	1667	737	44.2	42	11	215	204	20.5	22.8	56.7
Methotrexate	2122	708	33.4	164	27	806	780	12.1	14.1	73.7
Metoprolol	1711	699	40.9	95	19	465	446	13.7	17.2	69.1
Metformin	1554	698	44.9	126	17	720	703	18.3	13.3	68.3
Hydrochlorothiazide	1484	665	44.8	133	21	520	499	15.9	13.5	70.5
Lorazepam	1413	658	46.6	67	14	364	350	14.7	19.3	66.0
Loperamide	1345	656	48.8	95	16	400	384	15.1	18.3	66.6
Paclitaxel	1309	651	49.7	63	16	166	150	16.1	19.5	64.4
Esomeprazole	1267	637	50.3	92	22	463	441	13.2	16.2	70.6
Tramadol	1399	633	45.2	70	12	416	404	18.8	18.6	62.6
Insulin	1355	627	46.3	65	9	381	372	23.6	16.7	59.6
Vitamin d, unspecified	1604	619	38.6	201	33	658	625	14.2	10.0	75.8
Salbutamol	1554	599	38.5	139	24	742	718	16.9	10.0	73.1
Folinic acid	1279	591	46.2	65	21	185	164	10.5	20.1	69.4
Prednisolone	1330	569	42.8	74	19	367	348	12.7	20.6	66.8
Gabapentin	1528	552	36.1	182	21	655	634	14.5	15.0	70.5
Lisinopril	1309	505	38.6	108	22	507	485	12.5	16.2	71.3
Fluticasone	1296	498	38.4	150	29	729	700	13.7	12.7	73.7
Azathioprine	1609	479	29.8	217	37	762	725	11.7	10.9	77.5
Doxorubicin	1339	474	35.4	24	10	106	96	20.5	35.2	44.3
Clozapine	1719	436	25.4	100	23	770	748	12.4	21.6	66.1

We further evaluated TTO heterogeneity at the ATC first-level class. Latency distributions differed significantly across classes (Kruskal–Wallis H = 860.69, df = 7, p < 1 × 10^-180^), but the overall effect size was modest (ε^2^ = 0.0184), underscoring the importance of clinically interpretable differences rather than p-values alone (Supplementary Table S8). In quantile-based modeling using “Others” as the reference, antineoplastic and immunomodulating agents (ATC L) showed the most delayed onset (median ratio 3.602, 95% CI 3.273–3.964; predicted median 222.3 days; +161.3 days vs. reference), followed by respiratory system drugs (ATC R; ratio 2.581, 2.003–3.326; +98.0 days) and blood and blood-forming agents (ATC B; ratio 1.919, 1.700–2.166; +57.0 days), with consistent FDR-adjusted significance (Supplementary Table S9; Supplementary Figure S8). Collectively, these results indicate meaningful differences in TTO profiles across therapeutic classes, with particularly prolonged latency for long-term immunomodulating regimens.

### 3.5 Cross-database validation between FAERS and JADER

To externally validate FAERS-derived disproportionality signals, we conducted a cross-database comparison with the Japanese JADER database using the same DIO case definition and analytical framework. The top 50 suspect drugs independently identified within the JADER database are detailed in Table 4. Among the primary FAERS Top 50 suspect drugs, 20 overlapping drugs had sufficient information to be evaluated in both databases and were included in the cross-database validation set (Table 5).

**TABLE 4:** Disproportionality analysis for DIO in JADER (Q1 2004–Q4 2024): top 50 suspect drugs (ROR and 95% CI).

Drug	Number of DIO reports	ROR	95% CI (ROR)	Signal (ROR)	Strong signal (ROR)
Magnesium oxide	755	2.11	1.96–2.27	Yes	No
Clozapine	458	15.28	13.89–16.80	Yes	Yes
Sennoside	388	2.49	2.25–2.75	Yes	No
Amlodipine besilate	354	0.92	0.83–1.03	No	No
Bevacizumab	314	2.18	1.95–2.43	Yes	No
Prednisolone	308	0.57	0.50–0.63	No	No
Lansoprazole	306	0.99	0.88–1.10	No	No
Fluorouracil	280	1.92	1.71–2.16	Yes	No
Famotidine	275	1.06	0.94–1.20	No	No
Oxaliplatin	262	2.19	1.93–2.47	Yes	No
Sodium picosulfate hydrate	261	6.04	5.34–6.84	Yes	Yes
Calcium levofolinate	242	2.44	2.15–2.77	Yes	No
Daikenchuto	241	7.38	6.49–8.39	Yes	Yes
Loxoprofen sodium hydrate	227	0.78	0.69–0.89	No	No
Aspirin	221	0.75	0.66–0.86	No	No
Irinotecan hydrochloride hydrate	221	2.66	2.33–3.04	Yes	No
Dexamethasone	206	1.11	0.97–1.28	No	No
Dexamethasone sodium phosphate	196	1.38	1.20–1.59	Yes	No
Furosemide	178	0.61	0.53–0.71	No	No
Paclitaxel	176	1.48	1.27–1.72	Yes	No
Carboplatin	174	1.04	0.89–1.21	No	No
Oxycodone hydrochloride hydrate	169	2.33	2.01–2.72	Yes	No
Mosapride citrate hydrate	169	2.29	1.97–2.67	Yes	No
Tegafur/gimeracil/oteracil potassium	166	1.91	1.64–2.22	Yes	No
Rebamipide	166	0.78	0.67–0.90	No	No
Barium sulfate	164	15.92	13.58–18.65	Yes	Yes
Nifedipine	156	1.23	1.05–1.44	Yes	No
Risperidone	153	2.26	1.93–2.65	Yes	No
Doxorubicin hydrochloride	149	1.86	1.58–2.18	Yes	No
Flunitrazepam	148	1.88	1.60–2.22	Yes	No
Sodium valproate	145	1.41	1.20–1.66	Yes	No
*Clostridium* butyricum preparation	145	1.79	1.52–2.11	Yes	No
Brotizolam	144	1.12	0.95–1.32	No	No
Olanzapine	143	2.79	2.37–3.29	Yes	No
Bortezomib	138	3.81	3.22–4.51	Yes	No
Granisetron hydrochloride	134	1.49	1.26–1.77	Yes	No
Cyclophosphamide hydrate	133	0.99	0.84–1.18	No	No
Cisplatin	131	1.00	0.84–1.19	No	No
Metformin hydrochloride	131	1.31	1.10–1.56	Yes	No
Esomeprazole magnesium hydrate	129	0.94	0.79–1.12	No	No
Pembrolizumab	128	0.70	0.59–0.83	No	No
Rabeprazole sodium	128	0.91	0.76–1.08	No	No
Sulfamethoxazole/Trimethoprim	126	0.58	0.49–0.69	No	No
Alfacalcidol	124	1.07	0.89–1.27	No	No
Tacrolimus hydrate	124	0.66	0.55–0.79	No	No
Acetaminophen	123	0.59	0.50–0.71	No	No
Quetiapine fumarate	122	2.25	1.88–2.69	Yes	No
Sitagliptin phosphate hydrate	122	1.52	1.27–1.82	Yes	No
Allopurinol	120	0.84	0.70–1.00	No	No
Nivolumab	119	0.54	0.45–0.65	No	No

**TABLE 5:** Cross-database comparison of DIO disproportionality signals between FAERS and JADER for overlapping suspect drugs (n = 20): logROR concordance and ΔlogROR.

Drug	FAERS_Number of DIO reports	FAERS_ROR	FAERS_95% CI (ROR)	FAERS_Signal (ROR)	JADER_Number of DIO reports	JADER_ROR	JADER_95% CI (ROR)	JADER_Signal (ROR)	Direction concordant	Both signal (ROR)	ΔlogROR (JADER-FAERS)
Acetylsalicylic acid	3336	0.78	0.75–0.80	No	166	1.91	1.64–2.22	Yes	No	No	0.896
Azathioprine	1658	2.66	2.53–2.79	Yes	40	1.00	0.73–1.36	No	No	No	−0.978
Capecitabine	1594	3.76	3.58–3.95	Yes	110	1.98	1.64–2.39	Yes	Yes	Yes	−0.641
Clozapine	1870	3.68	3.52–3.85	Yes	458	15.28	13.89–16.80	Yes	Yes	Yes	1.424
Dexamethasone	2326	1.43	1.38–1.49	Yes	206	1.11	0.97–1.28	No	Yes	No	−0.253
Fluorouracil	1865	4.67	4.46–4.89	Yes	280	1.92	1.71–2.16	Yes	Yes	Yes	−0.889
Folinic acid	1356	4.29	4.07–4.53	Yes	42	1.76	1.30–2.38	Yes	Yes	Yes	−0.891
Furosemide	2210	0.92	0.88–0.96	No	178	0.61	0.53–0.71	No	Yes	No	−0.411
Gabapentin	1599	0.82	0.78–0.86	No	20	1.68	1.08–2.61	Yes	No	No	0.717
Insulin	1433	0.62	0.59–0.65	No	18	0.88	0.55–1.39	No	Yes	No	0.350
Lorazepam	1511	1.18	1.13–1.24	Yes	42	1.32	0.98–1.79	No	Yes	No	0.112
Methotrexate	2214	0.70	0.67–0.73	No	107	0.38	0.32–0.46	No	Yes	No	−0.611
Omeprazole	2880	1.12	1.08–1.17	Yes	67	1.01	0.79–1.28	No	Yes	No	−0.103
Ondansetron	2246	1.96	1.88–2.04	Yes	7	1.48	0.70–3.11	No	Yes	No	−0.281
Oxaliplatin	1619	4.58	4.36–4.81	Yes	262	2.19	1.93–2.47	Yes	Yes	Yes	−0.738
Paclitaxel	1352	2.65	2.51–2.80	Yes	176	1.48	1.27–1.72	Yes	Yes	Yes	−0.583
Paracetamol	5134	0.90	0.87–0.92	No	123	0.59	0.50–0.71	No	Yes	No	−0.422
Prednisolone	1405	1.26	1.19–1.32	Yes	308	0.57	0.50–0.63	No	No	No	−0.793
Sodium chloride	1826	1.71	1.63–1.79	Yes	13	0.94	0.54–1.61	No	No	No	−0.598
Vitamin b9	1861	0.86	0.82–0.90	No	43	0.68	0.50–0.91	No	Yes	No	−0.235

Overall, logROR point estimates showed moderate concordance between FAERS and JADER (Spearman r = 0.63, p = 0.003), with the LOESS trend indicating an overall positive monotonic relationship across drugs (Figure 2). Several leading signals remained directionally consistent (ROR >1) in both databases, supporting external robustness of the primary FAERS findings. However, effect sizes were not identical across systems. A ΔlogROR analysis demonstrated drug-level heterogeneity in signal magnitude, with some agents exhibiting higher disproportionality in JADER and others showing stronger signals in FAERS (Table 5; Figure 3). Collectively, these results suggest that cross-database validation supports the reproducibility of key signals while also highlighting meaningful database-specific variation in signal strength.

### 3.6 Comparison with product information

To contextualize pharmacovigilance signals with regulatory information, we reviewed the most recent product information for the 50 FAERS study drugs and classified label evidence for intestinal-obstruction–related terms (e.g., intestinal/bowel obstruction, ileus/paralytic ileus, pseudo-obstruction) as Yes/No/Unclear (Supplementary Table S6; Supplementary Figure S6). Overall, 12/50 (24.0%) drugs explicitly mentioned intestinal obstruction or closely related terms, 28/50 (56.0%) did not, and 10/50 (20.0%) were classified as Unclear due to non-specific wording (e.g., severe constipation or fecal impaction without explicit obstruction terminology) (Supplementary Table S6). Labels were predominantly sourced from the FDA (40/50, 80.0%), with the remainder obtained from other publicly available regulatory sources; all were accessed on 4 February 2026 (Supplementary Table S6).

When compared with FAERS disproportionality results, label evidence only partially overlapped with signal-positive drugs. Among the 28 drugs with positive FAERS signals by ROR, 10 (35.7%) had explicit label support, 7 (25.0%) were Unclear, and 11 (39.3%) had no intestinal-obstruction–specific wording (Table 1; Supplementary Table S6). Drugs showing both positive FAERS signals and explicit label mention included bevacizumab, clozapine, infliximab, loperamide, macrogol, morphine, ondansetron, oxaliplatin, paclitaxel, and potassium-containing preparations (Table 1; Supplementary Table S6). Conversely, two drugs with explicit label mention (oxycodone and tramadol) did not demonstrate positive FAERS disproportionality signals (Table 1), indicating that label wording may reflect broader class-level gastrointestinal hypomotility concerns rather than drug-specific excess reporting in FAERS.

Notably, several drugs with clear FAERS signals lacked specific intestinal-obstruction wording in their current product information. This group included high-signal anticancer and immunomodulating agents such as fluorouracil, capecitabine, folinic acid, vedolizumab, adalimumab, azathioprine, and doxorubicin (Table 1; Supplementary Table S6). Among them, capecitabine, fluorouracil, and folinic acid also demonstrated positive signals in JADER (Table 5), supporting cross-database robustness and suggesting that intestinal-obstruction risk may be under-recognized or inconsistently represented in existing product information for selected agents. This partial overlap between disproportionality signals and label evidence motivates further discussion on potential sources of discordance and implications for pharmacovigilance.

## 4 Discussion

Our study provides an integrated pharmacovigilance map of drug-induced intestinal obstruction (DIO) using FAERS (Q1 2004–Q4 2024) and independent external comparison in JADER. In the deduplicated FAERS reporting universe (18,532,100 unique reports), we identified 62,919 DIO cases and first summarized their demographic and clinical seriousness profiles, followed by systematic disproportionality screening using both frequentist (ROR) and Bayesian (IC) metrics for the 50 most frequently implicated suspect drugs. Several agents showed pronounced signals, including bevacizumab as the strongest association by ROR/IC and adalimumab as the largest contributor by report volume, highlighting that DIO signals cluster in specific therapeutic areas rather than being uniformly distributed across commonly used medications. Importantly, to reduce concerns that a Top-50 presentation might over-represent frequently used drugs or obscure heterogeneity, we complemented the primary analyses with prespecified sensitivity and stratified evaluations (e.g., functional vs. mechanical obstruction definitions, serious-outcome strata, demographic strata, and oncology vs. non-oncology indication stratification), and we further examined a broader all-drug threshold set to assess the robustness of the overall signal landscape. Finally, cross-database comparison in the overlapping drugs between FAERS and JADER showed only moderate concordance, underscoring that replicated signals should be interpreted as stronger hypotheses while discordant estimates may reflect differences in populations, prescribing patterns, co-medication structures, and reporting behaviors rather than definitive evidence for or against causality.

Over the 21-year study period, the annual number of reported DIO cases in FAERS increased steadily, rising from 941 reports in 2004 to 5,113 reports in 2024. This temporal increase likely reflects a combination of factors, including expansion of post-marketing surveillance, changes in reporting practices and awareness, and shifts in drug utilization and patient complexity over time, and should not be interpreted as a direct measure of incidence. Nevertheless, the observed pattern broadly aligns with the substantial and growing population-level burden of intestinal obstruction and paralytic ileus reported in contemporary burden-of-disease analyses, particularly among ageing populations in high-income settings (Long et al., 2023; Proctor et al., 2023; Han et al., 2025; Zhang et al., 2025; Zhao et al., 2025). In the subset of DIO cases with completely documented age and sex information (n = 43,025), adults aged 18–65 years constituted the largest group, followed by those aged 66–85 years, whereas reports in children and the very elderly were comparatively infrequent. Such an age pattern is consistent with clinical and epidemiologic literature indicating that middle-aged and older adults bear the greatest burden of intestinal obstruction and are also more susceptible to serious adverse drug reactions due to multimorbidity, polypharmacy, and age-related physiologic changes (Davies and O’mahony, 2015; Zazzara et al., 2021; McGettigan et al., 2024). The relatively lower reporting frequency in younger patients and those >85 years of age may therefore reflect differences in underlying exposure opportunities, comorbidity profiles, healthcare utilization, and reporting behaviors, as well as potential under-recognition of DIO in these populations. Further work is needed to disentangle how age, underlying diseases, and treatment patterns interact to shape DIO risk and to identify patient subgroups that might benefit from targeted monitoring and preventive strategies.

Notably, DIO reports were frequently associated with serious clinical outcomes. Among cases with available outcome information (n = 62,110), hospitalization was reported in 45,410 (73.1%) and death was reported in 8,191 (13.2%). These FAERS outcome categories are not mutually exclusive and do not establish causality, and they likely reflect preferential reporting of severe events and underlying clinical complexity rather than event-specific fatality attributable to DIO. Nevertheless, the predominance of hospitalization and the substantial proportion with reported death are consistent with prior clinical series showing considerable morbidity and mortality among patients with intestinal obstruction (Soressa et al., 2016; Atalay et al., 2021; Long et al., 2023), underscoring that DIO represents a clinically consequential adverse drug reaction with meaningful implications for healthcare utilization. Coupled with the predominantly delayed-onset patterns observed for several long-term immunomodulating regimens in our TTO analyses, these seriousness profiles support sustained vigilance beyond the early treatment period, rather than monitoring limited only to treatment initiation (Budnitz et al., 2011; Davies and O’mahony, 2015; Oscanoa et al., 2017).

Interestingly, we observed a modest female predominance among DIO case reports with recorded sex. In the subset with complete age–sex documentation (n = 43,025), females accounted for 52.8% of reports, males for 46.1%, and sex was coded as unknown in 1.1%. This pattern contrasts with several hospital-based series of acute intestinal obstruction, which have typically reported a higher proportion of male patients (Soressa et al., 2016; Atalay et al., 2021; Long et al., 2023). However, sex distributions in spontaneous-reporting datasets should be interpreted cautiously because they lack sex-specific exposure denominators and may reflect differential drug utilization, underlying disease mix, healthcare-seeking behavior, and reporting practices rather than true biological susceptibility. Women more frequently report constipation and other functional bowel symptoms in population-based studies, which could increase the likelihood of obstruction-like presentations in the presence of additional pharmacologic triggers (Verkuijl et al., 2020; Narayanan et al., 2021). In addition, sex- and gender-related differences in pharmacokinetics and pharmacodynamics—such as body composition, hormone milieu, and drug-metabolizing enzyme activity—may influence susceptibility to adverse drug reactions (Zucker and Prendergast, 2020). Multiple pharmacovigilance studies have also shown that adverse drug reactions are reported more often in women than in men, which could amplify a female predominance in FAERS even when underlying risks are similar (de Vries et al., 2019; Watson et al., 2019). Therefore, the observed female predominance should be viewed primarily as a signal of potential heterogeneity and reporting/exposure differences, and future studies using longitudinal healthcare data with sex-specific denominators are needed to clarify whether sex-stratified risks and mechanisms differ for DIO. In our stratified disproportionality analyses, most leading signals remained directionally consistent across sex and age strata, with only limited drug-level variation.

Geographically, DIO case reports in FAERS were concentrated in high-income countries, with the United States contributing the largest share (27,963; 44.5%), followed by Canada (6,824; 10.8%), Japan (5,575; 8.9%), France (2,505; 4.0%), and Germany (2,464; 3.9%), while the remaining 17,588 (27.9%) were distributed across other countries or regions. This pattern should be interpreted as a reporting distribution rather than a proxy for global incidence. FAERS is U.S.-anchored, and the volume and completeness of reports are influenced by national pharmacovigilance infrastructure, diagnostic and coding practices, drug availability, and regulatory/reporting incentives; therefore, regions with lower reporting capacity may be systematically under-represented despite a substantial clinical burden of intestinal obstruction. These geographic imbalances reinforce the need to interpret disproportionality signals as hypothesis-generating and to triangulate findings using complementary data sources and settings, including independent national databases, longitudinal healthcare data, and context-specific pharmacoepidemiologic studies.

Overall, the increase in annual DIO reports over time, together with the frequent reporting of serious outcomes in FAERS, underscores that DIO is a clinically consequential and resource-intensive adverse drug reaction. Our observations are consistent with surgical series showing that intestinal obstruction is associated with substantial morbidity and mortality (Soressa et al., 2016; Atalay et al., 2021), and they align with pharmacoepidemiologic evidence that adverse drug reactions account for a meaningful share of emergency hospitalisations, particularly among older adults (Budnitz et al., 2011; Oscanoa et al., 2017). However, trends and outcome distributions in spontaneous reporting systems are shaped by reporting behaviors, exposure opportunities, and case complexity, and should not be interpreted as direct measures of incidence or attributable risk. Accordingly, our findings are best viewed as a structured set of safety hypotheses that supports targeted clinical awareness and monitoring and motivates confirmation in longitudinal pharmacoepidemiologic studies and mechanistic investigations.

Among the Top 50 suspect drugs, we observed a clinically relevant discordance between disproportionality signals and current product information. When we compared FAERS signal detection with label evidence, only a subset of signal-positive drugs explicitly mentioned intestinal-obstruction–related terms, whereas several drugs meeting prespecified FAERS signal criteria still lacked obstruction-specific wording or were described only with non-specific motility/constipation language. In particular, multiple high-signal antineoplastic and immunomodulating agents—such as fluorouracil, capecitabine, folinic acid, vedolizumab, adalimumab, azathioprine, and doxorubicin—showed clear disproportionality in FAERS yet did not uniformly carry explicit obstruction terminology in available labels. For fluoropyrimidine-based regimens, a biologically plausible pathway is severe small-bowel toxicity (mucositis, ulceration, ileitis/enteritis) that may progress to ileus or obstruction in susceptible patients (Fata et al., 1999; Brignoli et al., 2023), and the observation that capecitabine, fluorouracil, and folinic acid also demonstrated positive signals in JADER supports cross-database robustness for these specific agents. At the same time, many of these drugs are preferentially used in patients with intra-abdominal malignancy or inflammatory bowel disease, populations with elevated baseline risks for bowel complications; therefore, signal–label discordance should be interpreted as a prioritization cue rather than proof of causality. Our oncology vs. non-oncology stratification and obstruction-subtype sensitivity analyses suggest that the overall signal landscape is not solely driven by a single diagnostic category or indication context, but residual confounding remains possible. Taken together, these findings support targeted clinical vigilance and structured follow-up evaluation (e.g., longitudinal pharmacoepidemiologic studies and mechanistic investigations) for selected high-signal drugs where explicit obstruction terminology may be inconsistently represented in current product information.

Antineoplastic and immunomodulating agents emerged as prominent categories among the highest-priority DIO signals in our analysis, consistent with the ATC-level enrichment observed for the Top 50 set. This pattern is biologically plausible given that these therapies can compromise intestinal mucosal integrity, vascular supply, and inflammatory homeostasis, thereby increasing susceptibility to ileus-like presentations and obstructive complications. Prior pharmacovigilance work using JADER similarly reported over-representation of cytotoxic and biologic therapies among drugs linked to gastrointestinal obstruction or perforation, supporting a class-level safety concern across settings (Satake et al., 2021). At the same time, these agents are preferentially used in populations with elevated baseline risks for bowel complications (e.g., intra-abdominal malignancy, inflammatory bowel disease, prior surgery, or advanced disease), and therefore confounding by indication remains a key interpretability constraint; notably, our oncology vs. non-oncology stratification suggested that major signals were generally detectable across strata while often appearing stronger within oncology-associated reports, which is consistent with both pharmacologic and clinical-context contributions. Beyond mucosal/vascular injury pathways, agents that markedly impair bowel motility also plausibly contribute to DIO risk. Clozapine can induce profound gastrointestinal hypomotility and severe constipation, providing a mechanistic pathway to ileus and obstruction (Every-Palmer et al., 2016), and chronic opioid therapy is a well-recognized cause of opioid-induced bowel dysfunction spanning constipation to pseudo-obstruction (Candy et al., 2022). In our case-definition sensitivity analyses, such motility-impairing agents tended to show comparatively stronger signals for functional ileus/pseudo-obstruction, whereas cytotoxic regimens more often aligned with mechanical-obstruction signals, reinforcing the importance of considering phenotype-specific heterogeneity when interpreting DIO signals. Collectively, these findings support targeted clinical vigilance for DIO in patients receiving long-term antineoplastic, immunomodulating, and strongly constipating therapies—particularly in settings of multimorbidity and polypharmacy—while underscoring the need for confirmation in longitudinal pharmacoepidemiologic studies that can account for indication and baseline bowel risk.

Time-to-onset (TTO) profiling adds a clinically interpretable temporal layer to the disproportionality findings by indicating when DIO tends to manifest after drug initiation (Fata et al., 1999; Farmer et al., 2018). Because individual FAERS case reports may list multiple co-prescribed suspect drugs with different therapy start dates, we evaluated TTO at the report–drug pair level. Among 106,660 DIO report–drug pairs for the Top 50 set, 54,371 lacked usable therapy start or event onset dates and 5,762 had implausible intervals (onset preceding therapy start or >3,650 days); therefore, 46,527 pairs (43.6%) were retained for downstream analyses. Across retained pairs, DIO exhibited a predominantly delayed-onset profile (median 100 days; IQR 19–469; P10–P90 3–1,459 days), indicating that many clinically relevant events accrue after weeks to months of exposure rather than being confined to treatment initiation. At the drug level, latency was heterogeneous, with median TTOs spanning an order of magnitude (e.g., 24 days for doxorubicin vs. 248 days for infliximab), and most agents still show a majority of events occurring beyond 30 days. Late-onset patterns were most prominent for biologics and immunomodulators (e.g., infliximab, adalimumab, vedolizumab, azathioprine), whereas comparatively earlier onset was observed for some cytotoxic agents, opioids, and corticosteroids (e.g., doxorubicin, fluorouracil, morphine, dexamethasone), consistent with distinct pharmacologic and clinical contexts of exposure. At the ATC first-level class, TTO distributions differed statistically, but the overall effect size was modest (ε^2^ = 0.0184), supporting emphasis on clinically interpretable contrasts rather than p-values alone (Scholl and van Puijenbroek, 2016); quantile-based modeling suggested the most delayed onset for antineoplastic and immunomodulating agents (ATC L), with smaller but consistent delays for selected other classes. From a practical standpoint, these latency profiles support differentiated vigilance: for drugs with relatively earlier onset patterns, clinicians should maintain a low threshold for evaluating evolving constipation, distension, and obstructive symptoms soon after initiation or dose escalation, whereas for biologics and long-term immunomodulators, monitoring should be sustained over prolonged therapy because delayed presentations predominate and may be insidious. Finally, TTO in spontaneous reports should be interpreted as supportive context rather than a causal timeline, given substantial missingness and potential imprecision of start/onset dates and the likelihood that reporting and coding practices differ across drugs and settings (Scholl and van Puijenbroek, 2016; Farmer et al., 2018; Satake et al., 2021).

Among the 20 prespecified overlapping drugs (defined as FAERS Top-50 drugs with ≥3 DIO reports in JADER after active-ingredient harmonisation), concordance between FAERS and JADER was moderate (Spearman r=0.63, p=0.003), supporting triangulation rather than definitive validation and limiting generalisability beyond the overlap set. Cross-database benchmarking provides an external check on the transportability of FAERS-based signals but also highlights heterogeneity driven by differences in drug availability and utilisation, case-mix and prescribing indications (including baseline bowel risk and oncology profiles), co-medication structures, diagnostic labeling, and reporting practices across systems. The ΔlogROR dumbbell plot further illustrates that some agents exhibit materially stronger signals in one database than the other; discordant estimates for drugs such as gabapentin and prednisolone are therefore best interpreted under non-mutually exclusive explanations, including context-specific effects, residual confounding/channeling, and differences in background exposure and reporting structures that can shift log-scale disproportionality estimates. Accordingly, cross-database comparisons should be viewed as triangulation rather than adjudication of causality: the most credible candidates for prioritisation are those combining strong within-database disproportionality, mechanistic plausibility, and at least directional consistency across settings, whereas discordant drugs warrant targeted, context-aware follow-up. Confirmatory analyses using longitudinal healthcare data (claims/EHR) with validated exposure windows, adjustment for indication and baseline bowel risk, and explicit modelling of co-medication patterns would help distinguish true population differences from reporting-system artefacts.

Several limitations should be acknowledged. First, FAERS and JADER are spontaneous reporting systems subject to under-reporting, differential/stimulated reporting, duplicate submissions, and incomplete or inconsistent fields; therefore, true incidence, absolute risk, and attributable fractions cannot be estimated, and disproportionality metrics should be interpreted as hypothesis-generating associations rather than causal effects. Second, outcome ascertainment relied on MedDRA PT–based definitions and reporter-coded suspect-drug roles; misclassification is possible, including overlap between ileus, pseudo-obstruction, and mechanical obstruction phenotypes, and FAERS outcome categories (e.g., hospitalization and death) are not mutually exclusive and may reflect reporting preference and case complexity rather than event-specific severity attributable to DIO.Third, residual confounding cannot be eliminated, particularly confounding by indication and disease severity (e.g., intra-abdominal malignancy/IBD), prior abdominal surgery, comorbidities, and co-medication patterns (opioids, antiemetics, other constipating agents), despite prespecified stratification and sensitivity analyses (including oncology vs. non-oncology and phenotype-based definitions). Fourth, selection and data-quality constraints may influence secondary analyses: the Top-50 focus improves estimate stability and interpretability but may not capture rare yet clinically important signals, and threshold-based “all-drug” sensitivity analyses mitigate but do not remove selection bias. Fifth, the time-to-onset analysis was restricted to report–drug pairs with complete and plausible start/onset dates, and such dates are frequently missing or imprecise; multiple suspect drugs within a report also introduce within-report dependence, so TTO findings should be viewed as supportive context rather than definitive latency estimates. Sixth, cross-database comparisons are constrained by limited drug overlap and by differences in coding practices, drug availability/utilization, and reporting behaviors across pharmacovigilance systems, which can produce discordant drug-level estimates. Finally, label comparison is inherently limited by regional and temporal variation in product information and terminology (including situations where labels describe upstream constipating effects without explicitly naming obstruction), and drug-name/ingredient harmonization in spontaneous reports may introduce additional non-differential misclassification that typically attenuates associations but can affect specific agents with ambiguous names. Although ingredient harmonisation improves comparability, residual misclassification may persist because drug names in spontaneous reports are often free text and may not reflect structured clinical medication records.

## 5 Conclusion

From FAERS reports spanning Q1 2004 to Q4 2024, with external benchmarking in JADER, we prioritised the 50 suspect drugs with the highest DIO report counts and characterised their disproportionality signals, robustness across prespecified sensitivity and stratified analyses, and time-to-onset profiles. Comparison with current product information suggested incomplete alignment between disproportionality signals and explicit obstruction terminology, highlighting drug–event pairs that merit closer post-marketing evaluation. Given that DIO reports were frequently associated with serious outcomes and that many events occurred after weeks to months of exposure, clinicians should maintain sustained vigilance when prescribing high-signal therapies, particularly in patients with elevated baseline bowel risk (e.g., older adults, prior abdominal surgery, or underlying bowel disease) and in the presence of constipating co-medications. These findings should be interpreted as hypothesis-generating rather than causal evidence, and priority signals warrant confirmation in longitudinal pharmacoepidemiological studies with validated exposure windows and rigorous confounder control, alongside mechanistic investigations to support risk-stratified prevention and management strategies.

## Data Availability

The original contributions presented in the study are included in the article/Supplementary Material, further inquiries can be directed to the corresponding author.

## Supplementary material

SUPPLEMENTARY TABLE S1 MedDRA Preferred Term (PT) cluster for drug-induced intestinal obstruction case definitions.

SUPPLEMENTARY TABLE S2 Sensitivity analysis across case-definition subtypes for the FAERS Top 50 drugs.

SUPPLEMENTARY TABLE S3 Serious outcome stratification (FAERS OUTC): disproportionality signals for death and hospitalization.

SUPPLEMENTARY TABLE S4 Age- and sex-stratified disproportionality analyses for the FAERS Top 50 drugs.

SUPPLEMENTARY TABLE S5 Oncology vs non-oncology stratified disproportionality analyses for the FAERS Top 50 drugs.

SUPPLEMENTARY TABLE S6 Regulatory label review for prioritized drugs: evidence classification (Yes/No/Unclear) and supporting text.

SUPPLEMENTARY TABLE S7 Selection-bias diagnostics for prioritizing Top 50 drugs in FAERS.

SUPPLEMENTARY TABLE S8 Time-to-onset (TTO) derivation and summary statistics at the report–drug pair level (FAERS).

SUPPLEMENTARY TABLE S9 Quantile regression (or prespecified TTO model) outputs for TTO: coefficients, uncertainty, and model diagnostics.

SUPPLEMENTARY TABLE S10 Drug–drug pair interaction metrics for intestinal obstruction: ΔlogROR, confidence intervals, and ranking.

SUPPLEMENTARY FIGURE S1 MedDRA PT terms used to define drug-induced intestinal obstruction case definitions.

SUPPLEMENTARY FIGURE S2 Heatmap of log(ROR) for the FAERS Top 50 drugs across alternative case definitions.

SUPPLEMENTARY FIGURE S3 Serious outcome stratification of disproportionality signals in FAERS (death and hospitalization).

SUPPLEMENTARY FIGURE S4 Age- and sex-stratified log (ROR) patterns for the FAERS Top 50 drugs.

SUPPLEMENTARY FIGURE S5 Oncology vs non-oncology stratified log (ROR) patterns for the FAERS Top 50 drugs.

SUPPLEMENTARY FIGURE S6 Label-review evidence matrix for prioritized drugs.

SUPPLEMENTARY FIGURE S7 Diagnostics for Top 50 prioritization under all-drug screening thresholds.

SUPPLEMENTARY FIGURE S8 Time-to-onset (TTO) distribution summaries for prioritized drug–event pairs in FAERS.

SUPPLEMENTARY FIGURE S9 Model-based evaluation of TTO heterogeneity (quantile regression or prespecified TTO model).

SUPPLEMENTARY FIGURE S10 Drug–drug pair analysis for intestinal obstruction: co-reported signals and interaction metrics.
