# Supplementary figures and images for: Cross-national pharmacovigilance of drug-induced intestinal obstruction: disproportionality signals in FAERS and external validation in JADER
Source: Front Pharmacol. 2026 Mar 20;17:1760208. doi: 10.3389/fphar.2026.1760208 (PMC13046714; doi:10.3389/fphar.2026.1760208)

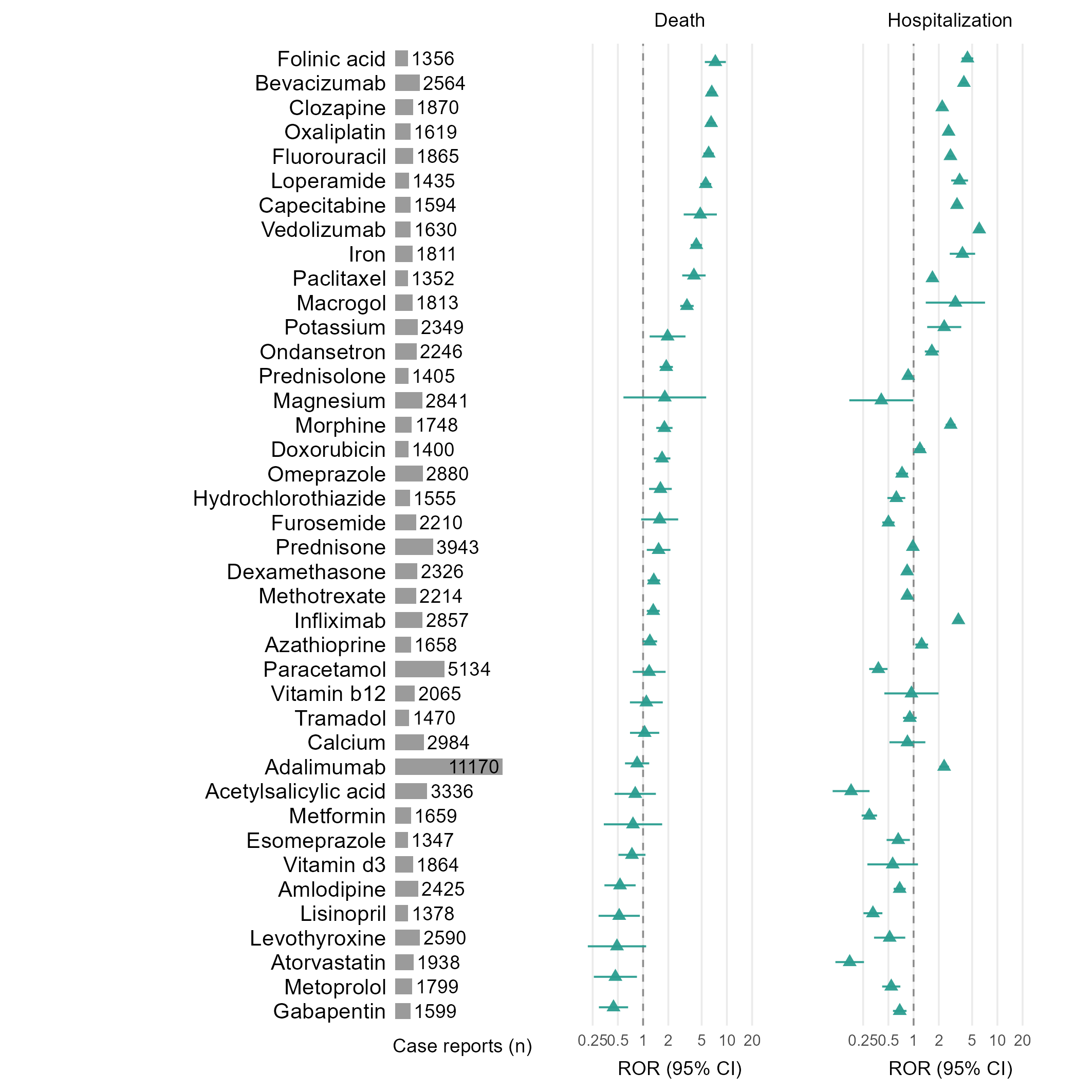

Supplement: Supplementary file 1 [file Image3.tiff]

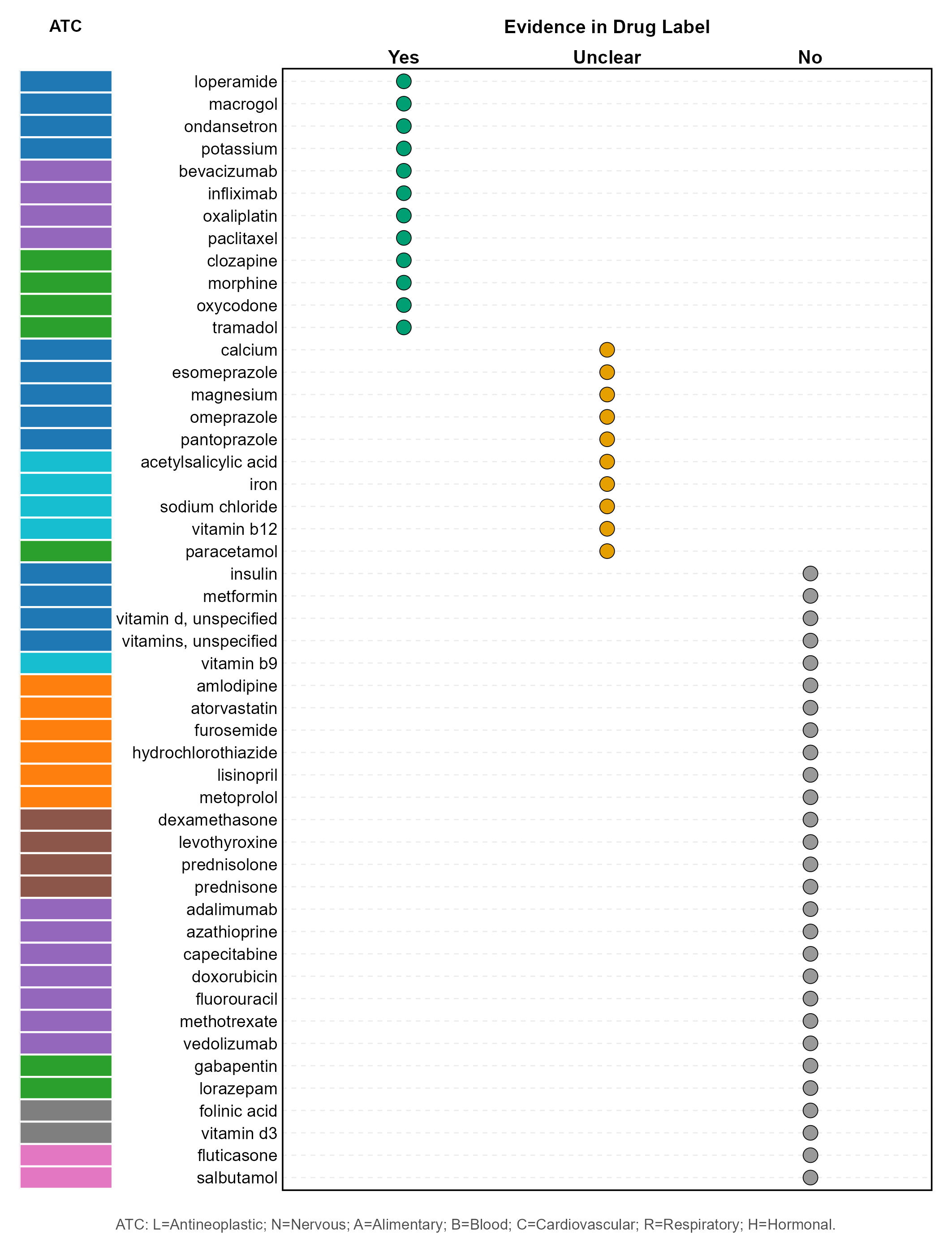

Supplement: Supplementary file 2 [file Image6.tif]

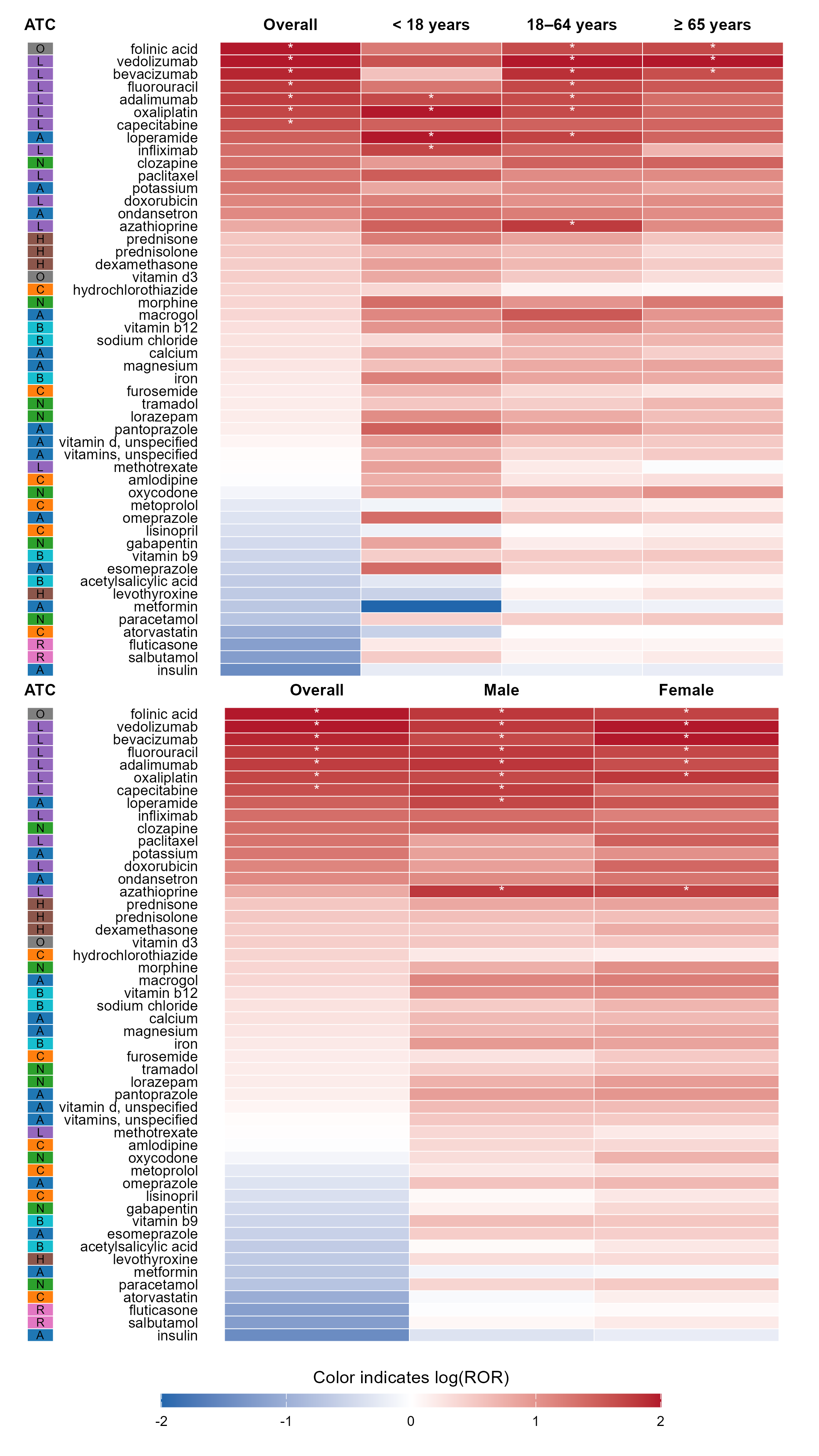

Supplement: Supplementary file 3 [file Image4.tif]

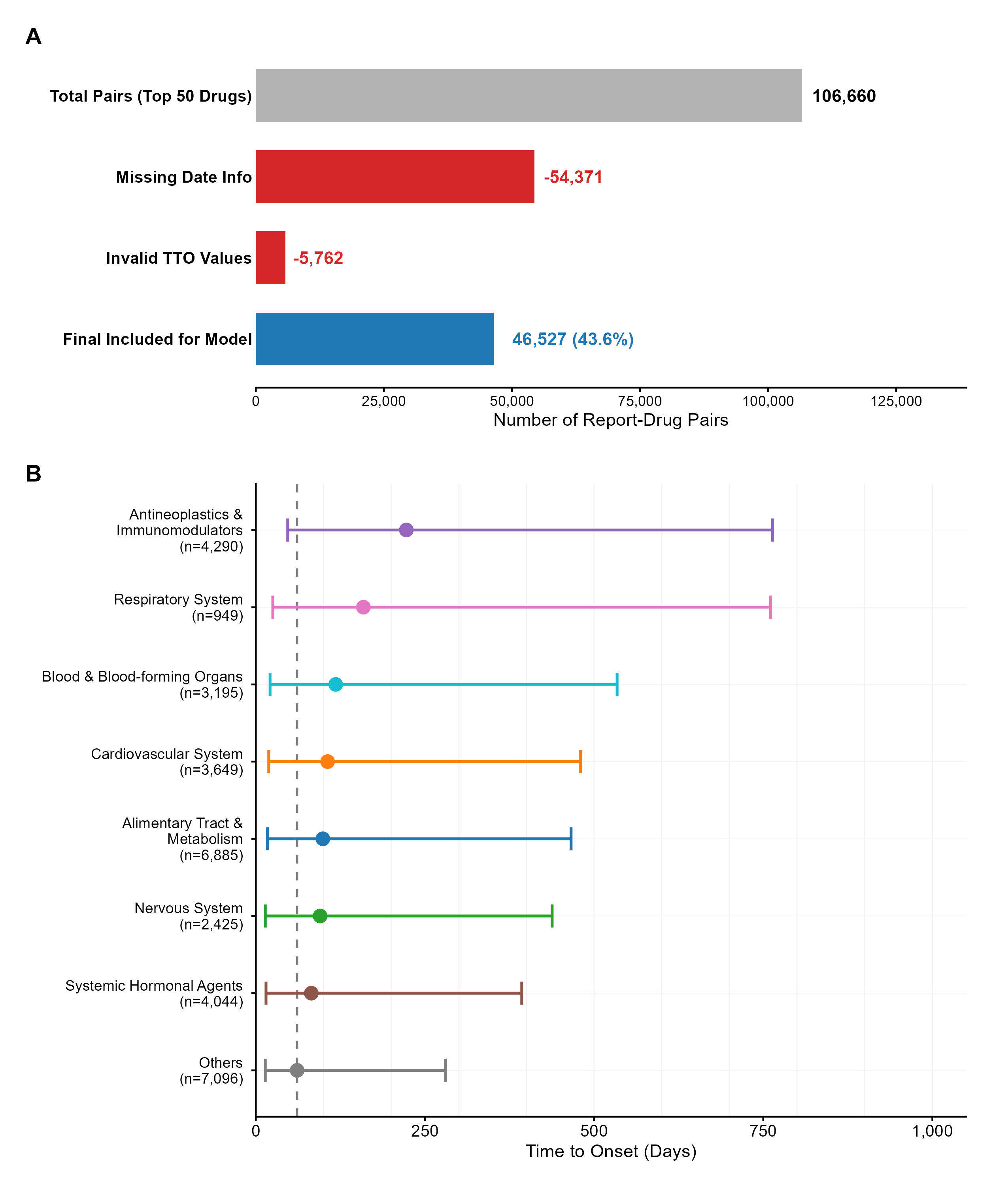

Supplement: Supplementary file 4 [file Image9.tif]

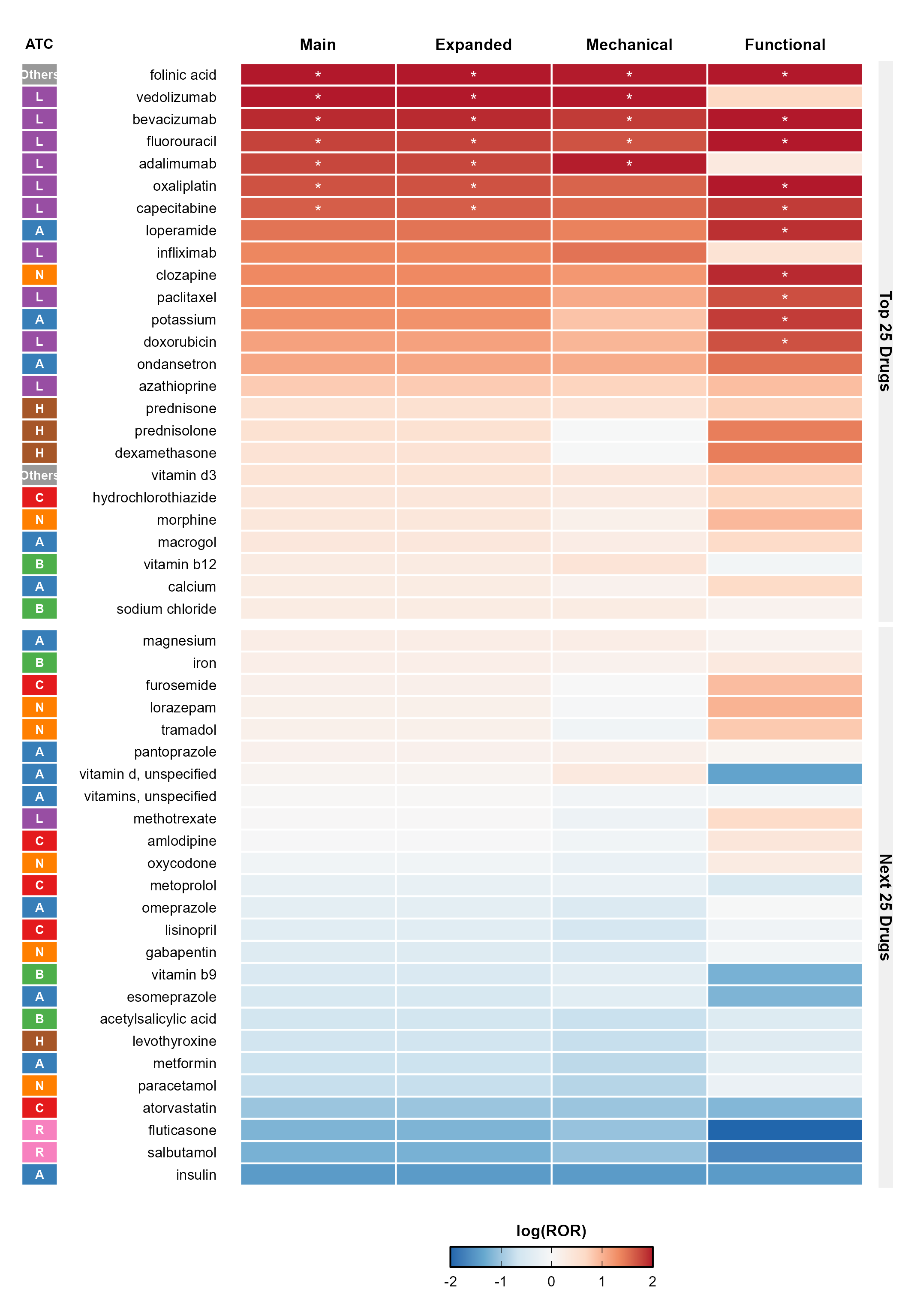

Supplement: Supplementary file 5 [file Image2.tif]

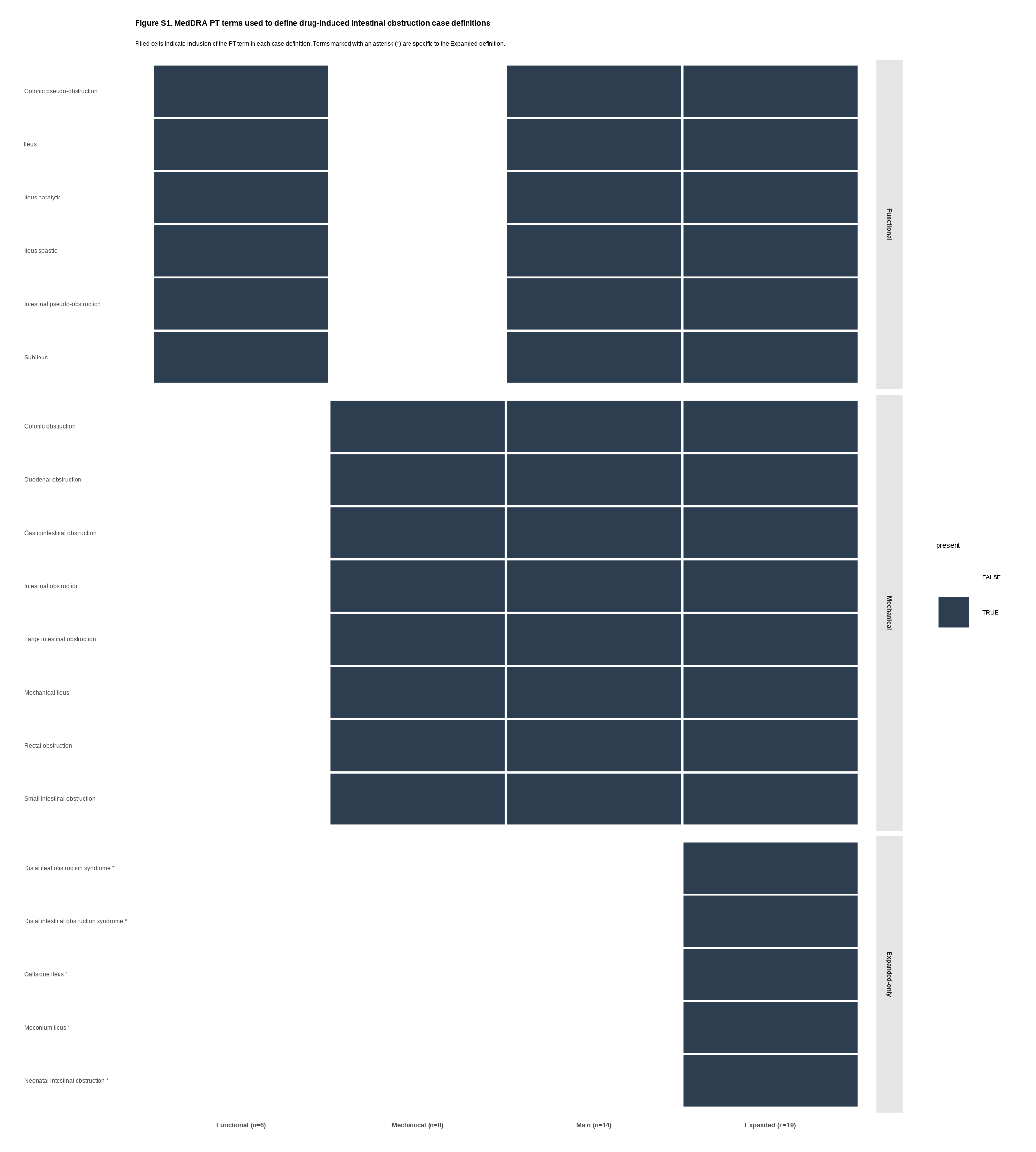

Supplement: Supplementary file 6 [file Image1.tif]

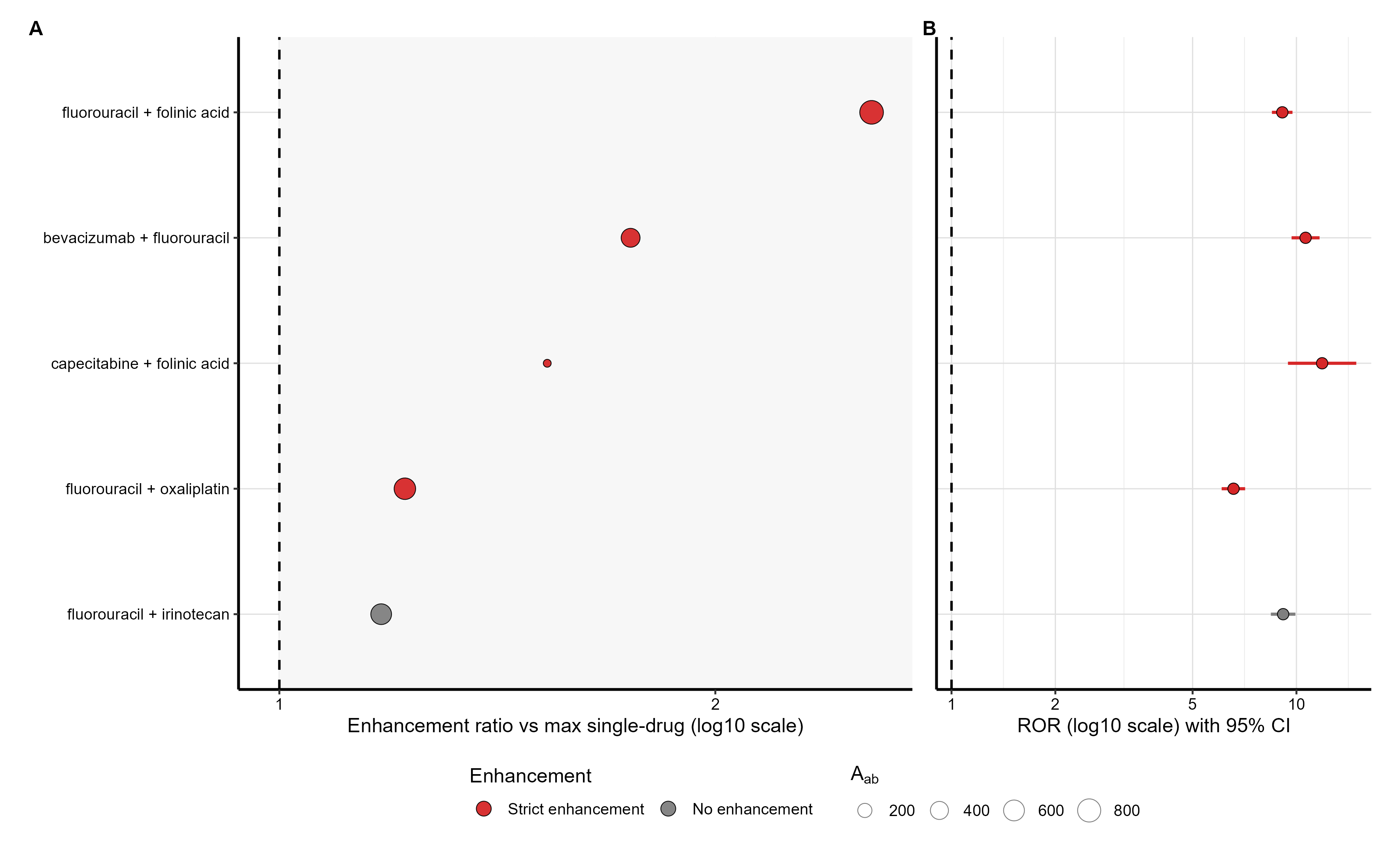

Supplement: Supplementary file 7 [file Image10.tif]

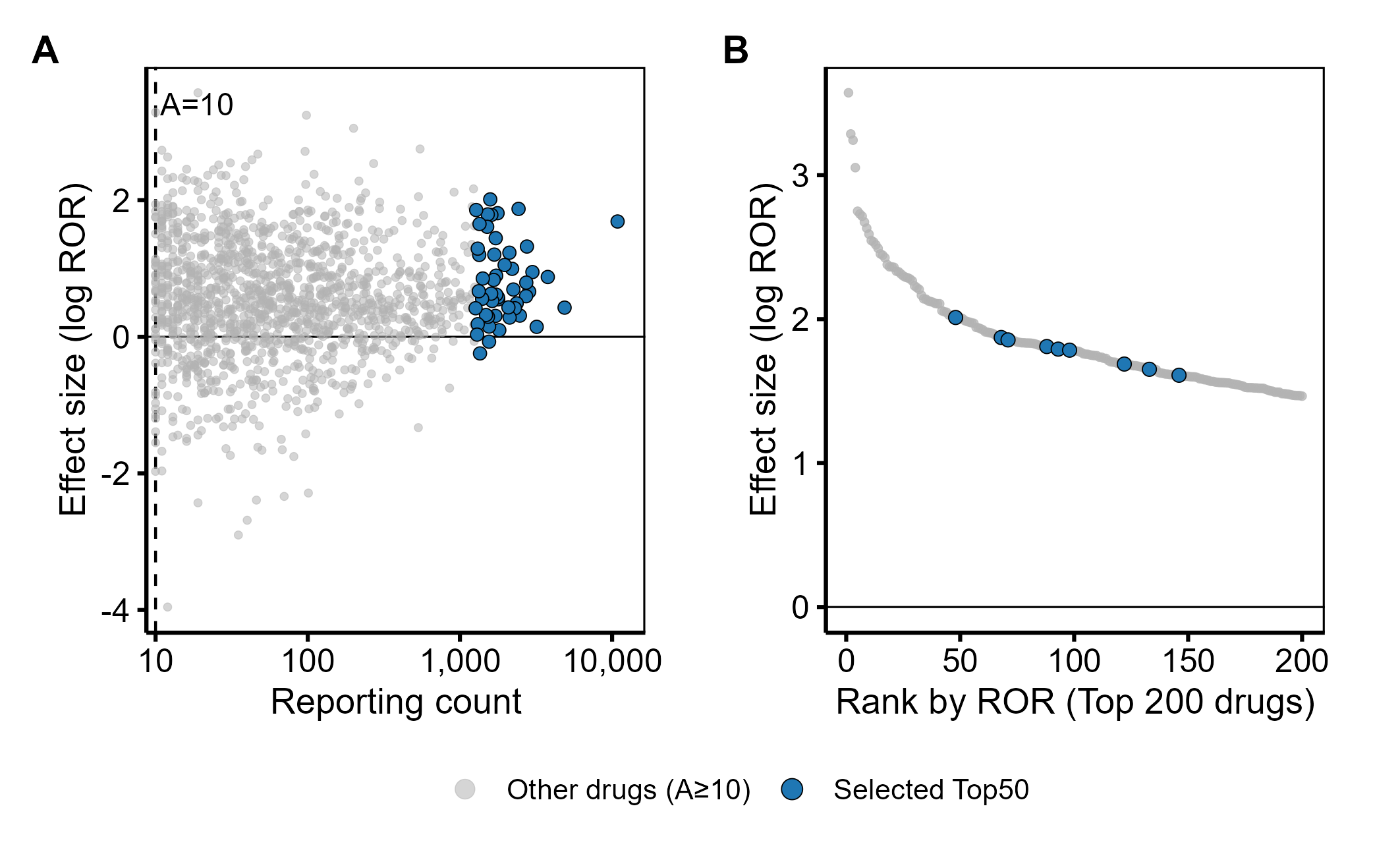

Supplement: Supplementary file 8 [file Image7.tif]

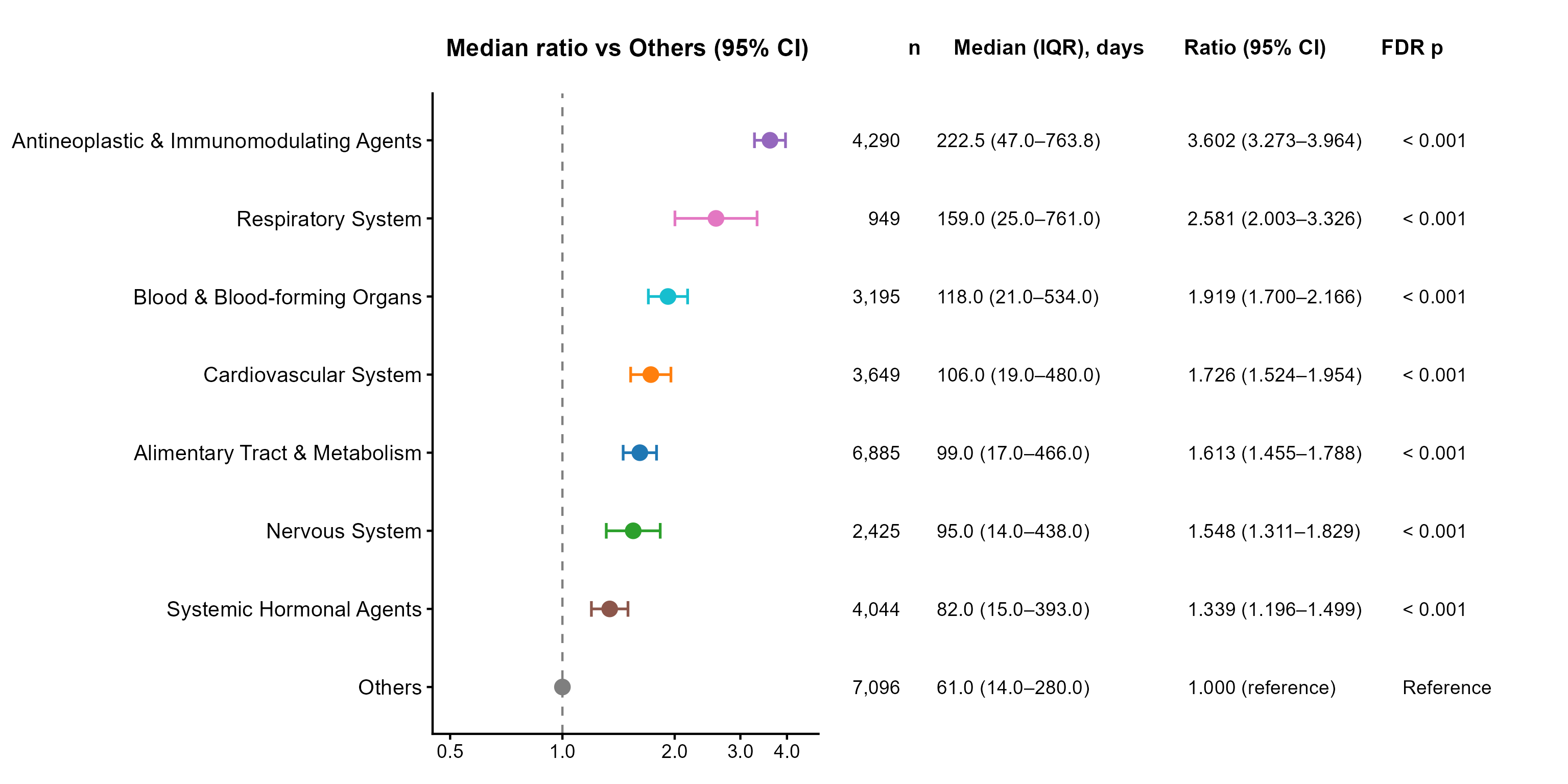

Supplement: Supplementary file 9 [file Image8.tif]

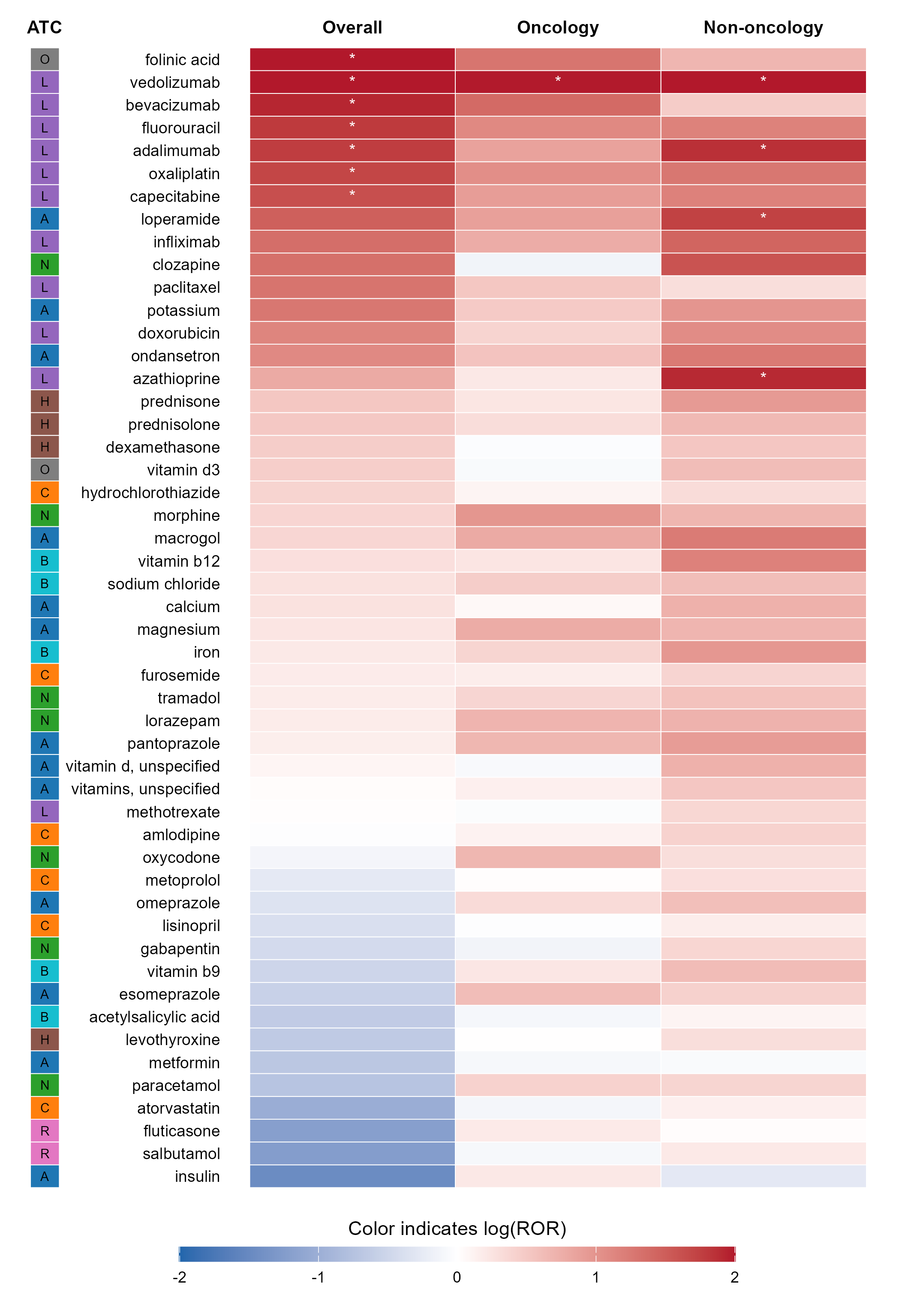

Supplement: Supplementary file 10 [file Image5.tif]
